# Cerebellar deep brain stimulation rescues Purkinje cell mitochondrial density in a genetic mouse model of cerebellar ataxia

**DOI:** 10.1016/j.brainresbull.2025.111704

**Published:** 2025-12-24

**Authors:** Lauren N. Miterko-Myers, Lauren E. Peacoe, Lita Duraine, Zhongyuan Zuo, Roy V. Sillitoe

**Affiliations:** a Department of Pathology and Immunology, Baylor College of Medicine, USA; b Department of Neuroscience, Baylor College of Medicine, USA; c Department of Pediatrics, Baylor College of Medicine, USA; d Department of Molecular and Human Genetics, Baylor College of Medicine, USA; e Program in Developmental Biology, Baylor College of Medicine, USA; f Development, Disease Models & Therapeutics Graduate Program, Baylor College of Medicine, USA; g Cerebellum Science Center, Texas Children’s Hospital, USA; h Jan and Dan Duncan Neurological Research Institute of Texas Children’s Hospital, 1250 Moursund Street, Suite 1325, Houston, TX 77030, USA

**Keywords:** Cerebellum, CAR8, Purkinje cell, Mitochondria, Endoplasmic Reticulum, Transmission Electron Microscopy, Deep Brain Stimulation

## Abstract

Deep brain stimulation (DBS) improves motor function in a growing list of movement diseases including Parkinson’s disease, dystonia, and tremor. There is evidence that DBS may also be effective in ataxia. It is not known why DBS is effective, but modulating cell activity and conferring neuroprotection are hypothesized to underlie its benefits. Understanding the effects of DBS on neurons is paramount to extending its clinical use in the treatment of various motor and non-motor diseases. Here, we stimulated the cerebellum of *Car8 waddles* (*Car8*^*wdl*^) mice, given the cerebellum’s important role in ataxia pathophysiology. Using transmission electron microscopy, we tested the effects of therapeutic neuromodulation on Purkinje cell subcellular structures, including the mitochondria and their proximity to the endoplasmic reticulum (ER). In the absence of stimulation, we found increased putative mitochondria-ER contacts in *Car8*^*wdl*^ Purkinje cells as well as mitochondrial size and density alterations. Low-frequency cerebellar DBS rescued mitochondrial density, but not size or putative contacts in *Car8*^*wdl*^ Purkinje cells. Although increased mitochondrial density and sustained ER contact are specific to DBS treatment, they do not determine efficaciousness. These data uncover a mode of intracellular plasticity in Purkinje cells after stimulation, enhancing our mechanistic understanding of DBS for cerebellar disorders.

## 1. Introduction

Deep brain stimulation (DBS) is an FDA-approved neurosurgical technique that is used to treat Parkinson’s disease (Limousin et al., 1998; Deuschl et al., 2006), tremor (Benabid et al., 1991), and dystonia (Vidailhet et al., 2005) through the intracranial delivery of electrical pulses to distinct nodes of the motor circuit (Kumar, 2002; Herrington et al., 2016). Given its robust effects and positive outcomes, there is growing interest to extend its use in the treatment of other motor and non-motor disorders (Lozano et al., 2019), such as epilepsy (Fisher and Velasco, 2014; Fisher et al., 2010), ataxia (Miterko et al., 2021; Teixeira et al., 2015; Georgiev et al., 2016), anxiety (Dijk et al., 2013; Li et al., 2022; Velasques et al., 2014), and depression (Velasques et al., 2014; Sheth et al., 2022). However, technical limitations of DBS, at least in part, prevent its widespread clinical use, which include: (1) the mechanisms of DBS are not clear and are likely specific to the symptom and stimulation target (Johnson et al., 2008; Rajamani et al., 2024); (2) there can be unwanted side effects that impact behavior (Frank et al., 2007; Yin et al., 2018; Zarzycki and Domitrz, 2020); and, (3) the benefits of stimulation can decrease over time (Limousin and Foltynie, 2019; Bulut et al., 2025; Peters and Tisch, 2021). While changing the stimulation target and frequency prolongs the functional effects of DBS (Miterko et al., 2021), determining whether intrinsic neuronal factors are also modulated with DBS will be important towards understanding the cellular prerequisites needed for the long-term suppression of symptoms.

DBS strategies that have traditionally targeted output nuclei in the thalamus (Vim, ventrointermediate nucleus) and basal ganglia (STN, subthalamic nucleus) show behavioral improvements due to neuronal desynchronization (Wilson and Moehlis, 2015), inhibition (Vitek, 2002), or activation (Miocinovic et al., 2006). The presence of intrinsic, neuroprotective effects after stimulation hint at its restorative potential (McKinnon et al., 2019), but the carryover effects reported after terminating DBS are short-lived (Grips et al., 2006; Beuter and Modolo, 2009). We hypothesize that a combination of intrinsic and extrinsic changes is needed to prolong the benefits of DBS, which likely occurs at an optimal frequency and at an optimal target. Through our previous optimization of DBS for the *Car8 waddles* (*Car8*^*wdl*^) mouse model of hereditary cerebellar ataxia, we found that stimulating the interposed cerebellar nuclei at beta-frequencies (13–20 Hz) during skilled exercise produced persistent benefits (Miterko et al., 2021). Among these benefits were long-lasting corrections to muscle firing and behavior, which indicate motor circuit repair (Miterko et al., 2021). However, it is unclear from these studies whether DBS alters intracellular processes to facilitate motor recovery.

Purkinje cells appear critical for circuit repair because inhibiting their neurotransmission prevented cerebellar DBS from improving motor behavior in mice (Miterko et al., 2021). Purkinje cell degeneration and misfiring are hallmark characteristics of ataxia (Koeppen, 1991; Hoxha et al., 2018), with new evidence suggesting that Ca^2+^ dysregulation (Egorova et al., 2023; Kasumu and Bezprozvanny, 2012), in conjunction with dysfunctional metabolic and secretory organelles (Liu et al., 2017; Ward et al., 2019; Mancini et al., 2019), are underlying causes and act by impairing transport, activating autophagy pathways, and altering depolarization probabilities (Kasumu et al., 2012; Walter et al., 2006; Brown and Loew, 2012; Wang et al., 2011; Li et al., 2025; Manolaras et al., 2023; Chakrabarti et al., 2009; Girard et al., 2012; Zhang et al., 2021). *Car8*^*wdl*^ mice exhibit several of these characteristics, including motor incoordination, Purkinje cell misfiring, and intracellular Ca^2+^ dysregulation (White et al., 2016; Miterko et al., 2019; Jiao et al., 2005; Hirasawa et al., 2005; Hirota et al., 2003), due to a loss-of-function mutation in the *Car8* gene. *Car8* is predominantly expressed in cerebellar Purkinje cells (Türkmen et al., 2009; Ali et al., 2012), where it encodes for a protein (CAR8) that competes with inositol triphosphate (IP3) in the binding of its receptor (IP3R1) (Hirota et al., 2003). Given that IP3R1 is largely localized to the endoplasmic reticulum and gates internal Ca^2+^ stores, loss of CAR8 is hypothesized to result in uncontrolled Ca^2+^ release into the cytosol and at inter-organelle membrane contact sites (Hirota et al., 2003; Ahumada-Castro et al., 2021). No studies to date have investigated the effects of Ca^2+^ dysregulation or DBS on organelle anatomy in *Car8*^*wdl*^ Purkinje cells.

Here, we used transmission electron microscopy (TEM) to characterize the intracellular architecture of *Car8*^*wdl*^ Purkinje cells, with and without cerebellar interposed DBS. We focused our studies on characterizing the mitochondria and their contacts with the endoplasmic reticulum (ER) of Purkinje cells for several reasons. First, mitochondria are highly dynamic structures that change in size, shape, and number in response to neuronal stresses, with Ca^2+^ dysregulation increasing mitochondria-ER interactions (Lee et al., 2018; Golic et al., 2014). Second, alterations in the frequency of these contacts or in the individual morphology of mitochondria impacts neuronal function by modulating neurotransmitter release and synaptic architecture (Fowler et al., 2019; Mironov and Symonchuk, 2006; Shirokova et al., 2020; Garcia et al., 2019; Cserép et al., 2018; Graham et al., 2017). Third, mitochondrial defects may be central to the pathogenesis of ataxia and many other motor diseases, including Parkinson’s disease (Vastegani et al., 2023; Banerjee et al., 2009), dystonia (Indelicato et al., 2024a, 2024b), and tremor (Kuo et al., 2012; Yoo et al., 2008). Fourth, recent *post-mortem* analyses in Parkinson’s disease patients show that mitochondrial properties in neurons of the substantia nigra improve in response to STN-DBS (Mallach et al., 2019; Chen et al., 2024). Therefore, we postulated that the genetic mutation in *Car8*^*wdl*^ mice likely affects the organization of mitochondria and the ER in Purkinje cells, which cerebellar DBS may rescue. Indeed, we found that there are fewer, but larger mitochondria in *Car8*^*wdl*^ Purkinje cells in addition to more putative mitochondria-ER contacts (MERCs) per mitochondrion. We also found that low-frequency cerebellar DBS—regardless of efficacy—rescues mitochondrial density, but not size or putative MERCs, suggesting the presence of differential subcellular responses, rather than total repair, in Purkinje cells after therapeutic stimulation.

## 2. Methods

### 2.1. Animals

*Car8*^*wdl*^ mutant mice (Stock #004625), and their C57BLKS/J control littermates, were originally purchased from The Jackson Laboratory (Bar Harbor, ME), then were maintained in our animal colony at Baylor College of Medicine (BCM). We used a standard PCR genotyping protocol to differentiate the mouse strains, with primers as previously described (White et al., 2016; Jiao et al., 2005). We determined their age by designating embryonic day (E) 0.5 as the day in which a vaginal plug was detected in pregnant female mice and postnatal day (P) 0 as the day in which the pups were born. All animals were aged to P80 with food and water provided *ad libitum*. For our study, 18 mice were used: 6 mice (N=3 C57BLKS/J control, N=3 *Car8*^*wdl*^) that did not receive surgery (‘untreated’), 6 mice (N=3 C57BLKS/J control, N=3 *Car8*^*wdl*^) that received surgery but no stimulation (‘sham’, ‘0 Hz’), and 6 mice (N=3 C57BLKS/J control, N=3 *Car8*^*wdl*^) that received surgery and stimulation (‘stimulated’, ‘20 Hz’). Both males and females were used. All experiments were performed under an approved IACUC animal protocol, which follows the institutional guidelines set forth by BCM.

### 2.2. Deep brain stimulation (DBS)

#### 2.2.1. Surgical procedure

Twelve of the 18 mice used in our study underwent surgery for DBS (N=6 C57BLKS/J control, N=6 *Car8*^*wdl*^). In preparation for surgery, the pedestals of two twisted bipolar electrodes (PlasticsOne; 0.127 mm width, 3.5 mm length) were soldered to position the stimulating electrodes 2.6 mm apart. Bondic, a UV-light activated bonding agent (Amazon), was used to adhere the soldered pedestals together. Then, 30 minutes prior to the start of the anesthesia, C57BLKS/J control and *Car8*^*wdl*^ mutant mice were provided with pre-operative analgesics (1.0 mg/kg Buprenorphine-SR, subcutaneous; 5.0 mg/kg meloxicam). During surgery, sedation was achieved and maintained with ~2–3 % isoflurane. Using sterile, antiseptic, stereotactic surgical techniques, the DBS electrode implants made were bilaterally inserted into the cerebellum to target the interposed cerebellar nuclei (Anterior-Posterior: −6.40 mm; Medial-Lateral: ±1.30 mm; Dorsal-Ventral: −2.50 mm), then secured in place with C&B Metabond (Parkell, Inc., Edgewood, NY, USA, SKU: S380) and Teets ‘Cold Cure’ Dental Cement (A-M Systems, LLC, Carlsborg, WA, USA, Catalog #525000 and #526000). All the surgically implanted mice were provided with a post-operative analgesic for 72 hours, during which time the mice were closely observed for full recovery.

#### 2.2.2. Stimulation procedure

The 6 mice that did not receive surgery (N=3 C57BLKS/J control, N=3 *Car8*^*wdl*^) remained untreated and did not undergo the following stimulation procedure. Of the 12 mice with the surgically implanted electrodes (N=6 C57BLKS/J control, N=6 *Car8*^*wdl*^), 6 were randomly assigned to experimental groups receiving sham (no DBS, 0 Hz; N=3 C57BLKS/J control, N=3 *Car8*^*wdl*^) and 6 to experimental groups receiving DBS (20 Hz; N=3 C57BLKS/J control, N=3 *Car8*^*wdl*^) treatment. All 12 mice, regardless of their assigned experimental group, were connected to stimulation equipment, consisting of a Master8 pulse generator and an Iso-Flex stimulus isolator (AMPI, Jerusalem, Israel), 96 hours post-operation (Beckinghausen et al., 2023). For the mice receiving sham treatment, the Master8-Iso-Flex system was never turned on. For the mice receiving stimulation, the Master8-Iso-Flex system was programmed and turned on to deliver 60 μs square biphasic electrical pulses at a current amplitude of 30 μA and a frequency of 20 Hz to the mouse cerebellum, as described in prior studies (Chiken and Nambu, 2016; Apetz et al., 2019; Koeglsperger et al., 2019; Miterko et al., 2021). We observed robust behavioral improvements in *Car8*^*wdl*^ mice through combining these parameters with rotarod exercise and therefore continued this pairing in the present study (Miterko et al., 2021). The current amplitude and pulse width were originally selected based on their routine delivery in micro- and macro-stimulation protocols, where neuronal activity in motor areas are altered with limited side effects using low currents (Rizzone et al., 2001; Chiken and Nambu, 2013; Histed et al., 2009; Rajan et al., 2015). The small size of our DBS target and its close proximity to the fastigial cerebellar nuclei further warranted parameters that would restrict stimulation to the interposed nuclei (Arcot Desai et al., 2014; Bagshaw and Evans, 1976; Joucla et al., 2012). DBS-treated mice received the same stimulation protocol and were not individually thresholded for consistency across studies and to limit confounding effects.

To exercise the sham and DBS-treated mice, we set the rotarod to accelerate from 4 to 40 rpm in 5 minutes (ENV-576M and ENV-571M, Med Associates, Inc., Vermont, USA) and ran the program for 8 days. Mice stayed on the rotarod for a maximum of 300 seconds per trial for a total of 4 trials per day. Ten minutes separated each trial. Latency to fall values were recorded on each day, then used to calculate DBS efficacy: $\%\text{improvement}=\left(\frac{\left(\text{Average latency to fall},\text{Days}\;8-11\right)-\left(\text{Average latency to fall},\text{Days}\;3-4\right)}{\text{Average latency to fall},\text{Days}\;3-4}\right)\times 100\%$.

During the first 4 days, mice in both treatment groups were connected to the Master8-Iso-Flex equipment for a 5-minute acclimation period before being introduced to the rotarod and while being exercised on the rotarod afterwards. No stimulation was given to any of the mice on these 4 days. After 3 days of rest, the same mice were reintroduced to the rotarod for the remaining 4 days while being stimulated (20 Hz DBS group) or not being stimulated (0 Hz sham group). Stimulation was provided for 5 minutes before the rotarod as well as during the rotarod, for a maximum of 5 minutes per trial (20 minutes per day). Sham and DBS-treated mice were perfused before tissue analysis after their eighth day on the rotarod, using the protocol described below. Untreated mice were perfused without surgery and rotarod exercise, also using the protocol described below.

### 2.3. Transmission electron microscopy (TEM)

#### 2.3.1. Perfusion and tissue preparation

All 18 mice were anesthetized with 2,2,2-tribromoethanol (Avertin) and transcardially perfused with 0.1 M Phosphate Buffered Saline (PBS), then Modified Karnovsky’s fixative containing 2 % paraformaldehyde and 2.5 % glutaraldehyde in 0.1 M PBS (pH 7.4) and buffered to 320 mmol/kg with sodium cacodylate. After perfusion, the cerebella were carefully dissected out, sliced into 1 mm sagittal sections, and placed into scintillator vials with fresh fixative for overnight storage on a rotator at 4°C. Tissue sections were then processed inside a Ted Pella Bo Wave Vacuum Microwave 3 days later and further post-fixed using 1 % osmium tetroxide. Post-fixed tissue was dehydrated through increasing concentrations of ethanol (30–100 %) and propylene oxide. Embed 812 resin was gradually introduced to the dehydrated tissue with propylene oxide under vacuum until the Embed 812-proylene oxide mixtures could be replaced with pure resin. The tissue was then embedded into regular Beem capsules, cured in a 62°C oven for 5 days, and sectioned onto grids at 50 nm. Grids were stained with 1 % uranyl acetate and lead citrate for 15 and 3 minutes, respectively.

#### 2.3.2. Image acquisition and quantification

Low- (1500–5,000x) and high-powered (10,000x) TEM images were captured using a JEOL JEM 1010 transmission electron microscope with an AMT XR-16 mid-mount 16 mega-pixel digital camera. Each low-powered (1500–5,000x) TEM image contained a whole Purkinje cell soma while each high-powered (10,000x) TEM image zoomed in on roughly one quadrant area (dependent on Purkinje cell soma size). In total, 1 low-powered (1500–5,000x) TEM image and 2–13 high-powered (10,000x) TEM images were taken from each Purkinje cell in each animal. At least 4 Purkinje cells were imaged per animal, resulting in 18 Purkinje cells imaged from 3 untreated C57BLKS/J control mice, 15 Purkinje cells imaged from 3 untreated *Car8*^*wdl*^ mice, 31 Purkinje cells imaged from 3 sham C57BLKS/J control and 3 sham *Car8*^*wdl*^ mice, and 30 Purkinje cells imaged from 3 stimulated C57BLKS/J control and 3 stimulated *Car8*^*wdl*^ mice. ImageJ software was used to quantify the number, size, and shape of individual mitochondria as well as the number of putative MERCs. All images were calibrated to scale in ImageJ prior to quantification.

The following criteria were used to identify organelles and MERCs in cerebellar Purkinje cells. Mitochondria were identified by their double membranes and the presence of cristae (Song et al., 1991). ER were identified by their tubulous morphology, membranes, and a ~20–30 nm diameter (Terasaki et al., 2013; Karagas and Venkatachalam, 2019). MERCs were defined by anatomical proximity. Previous electron tomography studies have determined that ER within 200 nm of individual mitochondria interact (Giacomello and Pellegrini, 2016). However, ER within 15–30 nm of individual mitochondria are connected by mitochondria-associated ER membranes (MAMs), which mediate ionic transfers, including the exchange of Ca^2+^ (Giacomello and Pellegrini, 2016; Csordás et al., 2006, 2018; Rizzuto et al., 1998; Raturi and Simmen, 2013). Mitochondria also contact both rough (ribo-MERCs) and smooth (MERCs) ER (Giacomello and Pellegrini, 2016). Given the proposed intracellular function of CAR8, we analyzed putative MERCs and ribo-MERCs with a cleft thickness of 30 nm or less in this study.

Using the “Analyze Particles” feature in ImageJ, cytoplasmic area (Cytoplasmic Area (nm^2^) = Area of the Purkinje Cell (nm^2^) – Area of the Nucleus (nm^2^)) and mitochondrial density (Mitochondrial Density (number/nm^2^) = Mitochondria number/Cytoplasmic area of the Purkinje Cell (nm^2^)) were calculated from low-powered (1500–5,000x) TEM images. The number of mitochondria and MERCs were manually counted from high-powered (10,000x) TEM images. For ER with several branches near the mitochondria, only one measurement was taken from the closest branch. MERC frequency was calculated using the following equation: MERC frequency = Total number of MERCs/Total number of mitochondria. We also analyzed whether MERCs occur on all mitochondria by quantifying their presence or absence. To quantify the shape of mitochondria, we used the “Shape Descriptors” function in ImageJ to calculate the area and aspect ratio (Aspect ratio = Major axis/Minor axis). Elongated mitochondria have aspect ratios significantly greater than 1 whereas circular mitochondria have aspect ratios close to 1 because the lengths of the major and minor axes are almost equivalent. Measurements from each image (~quadrant) were combined to get an average for each Purkinje cell. Averages were plotted in bar and contingency graphs.

### 2.4. Data summaries and statistical analyses

A summary of the data and statistical tests used in this study are in Table 1. Data are reported as the mean ± SEM and were analyzed from individual Purkinje cells after statistically determining sample independence using *a priori* intra- and inter-variability calculations (Table 1) (van der Heijden et al., 2022). Two-tailed, unpaired Student’s *t*-tests (p<0.05) were used to compare cytoplasmic areas, total MERC numbers, MERC frequencies, as well as the size, shape, and density of mitochondria in Purkinje cells from untreated (no surgery) C57BLKS/J control and *Car8*^*wdl*^ mice. Mitochondrial size, shape, and density in sham (0 Hz) or DBS-treated (20 Hz) C57BLKS/J control and *Car8*^*wdl*^ Purkinje cells were compared using two-way ANOVAs (p<0.05) after data were normalized according to previously published methods (Normalized Value = (Raw Mitochondrial Area, Aspect Ratio, or Density / Untreated (No Surgery) C57BLKS/J Control Mitochondrial Area, Aspect Ratio, or Density) * 100 %) (Mallach et al., 2019). *Post hoc* analyses were performed with Tukey’s multiple comparisons tests when significant main effects were observed. Chi-squared tests (p<0.05) were conducted to compare the presence or absence of MERCs on C57BLKS/J control and *Car8*^*wdl*^ mitochondria within Purkinje cells of all experimental groups. Correlation matrices using Pearson *r* coefficients were generated to determine whether *Car8*^*wdl*^ Purkinje cells exhibited patterned intracellular compositions after different treatment regimens. K-means clustering in RStudio (Version 2025.09.2+418) then grouped sham and DBS-treated *Car8*^*wdl*^ Purkinje cells based on similarities in treatment, treatment efficacy, mitochondrial density, mitochondrial area, total MERC number, and MERC frequency. A two-way ANOVA with Tukey’s multiple comparisons tests was employed again to compare group means. All statistical tests were run in Prism8 Software (Version 10.1.0, 264).

## 3. Results

### 3.1. Increased frequency of putative mitochondria-ER contacts in Car8^wdl^ Purkinje cells

**Fig. 1.**
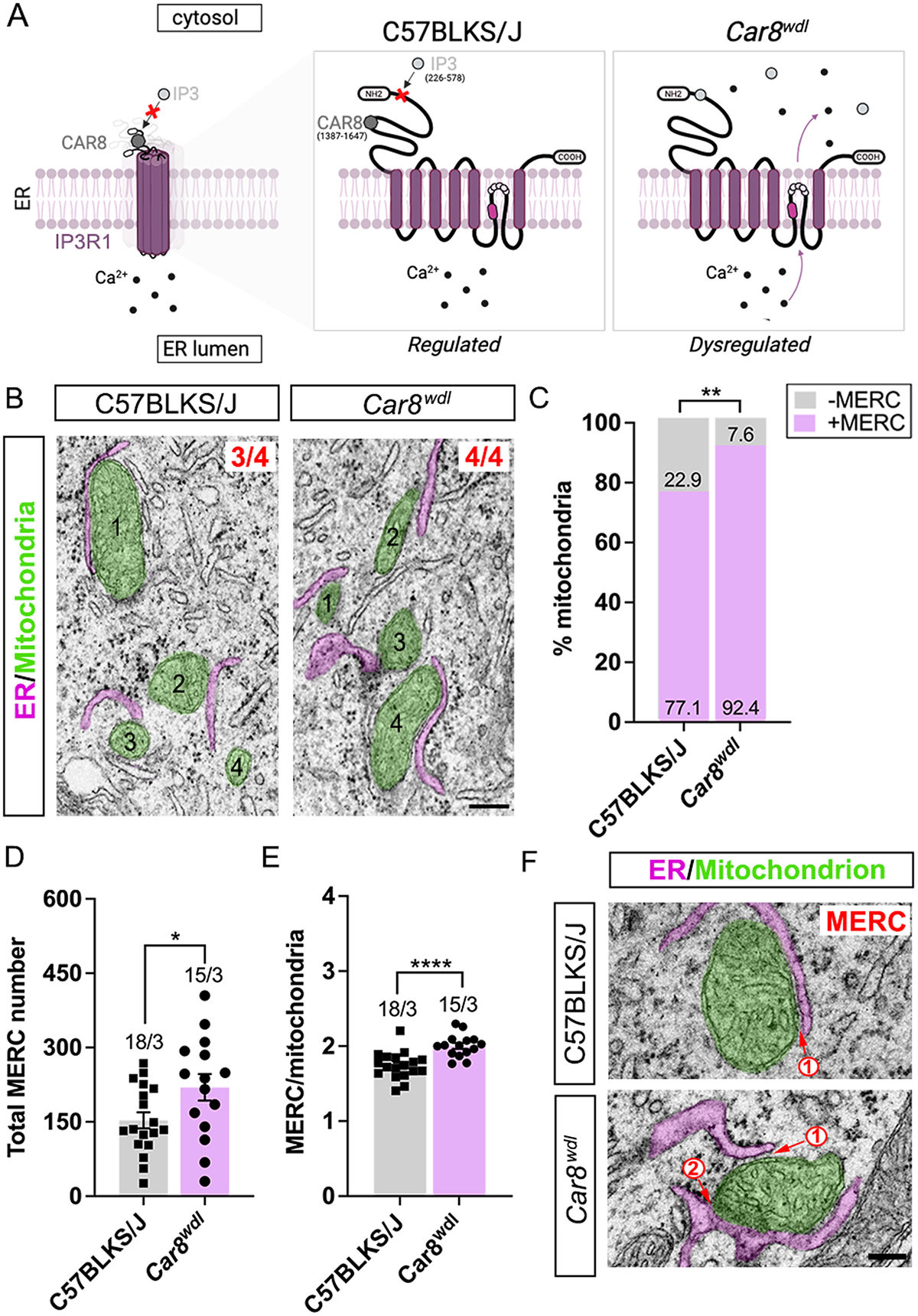
Loss of *Car8* from cerebellar Purkinje cells increases MERC frequency. **A.** Schematics of a monomer comprising the IP3R1 receptor and its hypothesized role with CAR8 in Ca^2+^ homeostasis. When CAR8 is present in the Purkinje cell, it binds IP3R1 at residues 1387–1647 (Hirota et al., 2003), which prevents IP3 from binding IP3R1 at residues 226–578 and regulates Ca^2+^ release (Yoshikawa et al., 1996). When CAR8 is absent from the Purkinje cell, IP3 freely binds to IP3R1, promoting dysregulated Ca^2+^ release into the cytosol. **B-C.** More mitochondria putatively contact ER (<30 nm) in *Car8*^*wdl*^ than in C57BLKS/J control Purkinje cells. Mitochondria are numbered and pseudo-colored **green** while ER are pseudo-colored **magenta**. One putative MERC is highlighted per mitochondrion. Numbers on TEM images denote the number of mitochondria associating with ER / total number of mitochondria in the field-of-view. Numbers on the contingency graph represent the percentage of mitochondria with no MERCs (**gray**) or at least 1 MERC (**magenta**) from 15 to 18 Purkinje cells across 3 C57BLKS/J control and 3 *Car8*^*wdl*^ mice. Scale bar measures 300 nm. **D.** *Car8*^*wdl*^ Purkinje cells have a greater total number of MERCs than C57BLKS/J control Purkinje cells. **E-F.** MERCs (**red** numbered arrows) more frequently associate with the same mitochondria in *Car8*^*wdl*^ Purkinje cells than in C57BLKS/J control Purkinje cells. Scale bar measures 150 nm. Numbers on bar graphs denote Purkinje cell number (n) / animal number (N), or 15 Purkinje cells from 3 untreated *Car8*^*wdl*^ mice and 18 Purkinje cells from 3 untreated C57BLKS/J control mice. Data analyzed using a chi-squared test (**C**) or unpaired two-tailed student’s t-test after testing for normality (**D-E**). * p<0.05; ** p<0.01; **** p<0.0001. Mean ± SEM. Schematic in (**A**) was created in BioRender (Miterko, L. (2025) https://BioRender.com/r4zvxr4) and published with permission.

CAR8 protein is predominantly expressed in Purkinje cells, where it is hypothesized to regulate IP3 binding to the IP3R1 receptor on the ER and subsequently Ca^2+^ release into the cytosol (Fig. 1A) (Hirota et al., 2003). If elevated levels of Ca^2+^ in the cytosol do not trigger cell apoptosis, then it can alter mitochondrial-ER interactions (Nicotera and Orrenius, 1998; Eisner et al., 2018; Urabe et al., 2020). We previously did not find cell degeneration after loss of *Car8* (White et al., 2016; Miterko et al., 2019). Therefore, we turned to TEM to determine whether a loss-of-function mutation in *Car8* causes intracellular changes within Purkinje cells, which might accompany the structural and circuit changes of cerebellar neurons (Hirasawa et al., 2005; Lamont and Weber, 2015; Miterko et al., 2019; Miterko and Sillitoe, 2018; White et al., 2016). For these studies, we performed TEM in untreated C57BLKS/J control and *Car8*^*wdl*^ mice and found that the mitochondria in *Car8*^*wdl*^ Purkinje cells more frequently contact ER than the mitochondria in C57BLKS/J control Purkinje cells (Fig. 1B–C). On average, 92.4 ± 0.2 % of mitochondria putatively contact ER in *Car8*^*wdl*^ Purkinje cells (n=15 Purkinje cells from 3 mice) versus 77.1 ± 8.3 % of mitochondria in C57BLKS/J control Purkinje cells (n=18 Purkinje cells from 3 mice; p=0.0060; Fig. 1C). To determine whether *Car8*^*wdl*^ mitochondria are more likely to interact with ER than C57BLKS/J control mitochondria because there may be more ER in *Car8*^*wdl*^ Purkinje cells, we quantified the total number of MERCs. Since only one MERC measurement was taken per ER, the total number of MERCs counted is equivalent to the number of ER present within 30 nm of each mitochondrion. We found that *Car8*^*wdl*^ Purkinje cells (n=15 Purkinje cells from 3 mice) have significantly more MERCs (219.7 ± 26.8) than C57BLKS/J control Purkinje cells (n=18 Purkinje cells from 3 mice; 153.1 ± 16.3 MERCs), indicating that *Car8*^*wdl*^ Purkinje cells contain more ER in proximity to their mitochondria (Fig. 1D; p=0.0352). The distribution of MERCs also significantly differs between C57BLKS/J control and *Car8*^*wdl*^ Purkinje cells. On average, C57BLKS/J control Purkinje cell mitochondria had 1.74 ± 0.04 MERCs while mitochondria in *Car8*^*wdl*^ Purkinje cells had 2.00 ± 0.04 MERCs (Fig. 1E–F; p<0.0001). Together, these data reveal abnormal mitochondria-ER associations in the CAR8-deficient cerebella.

### 3.2. Car8^wdl^ Purkinje cells contain fewer but larger mitochondria

**Fig. 2.**
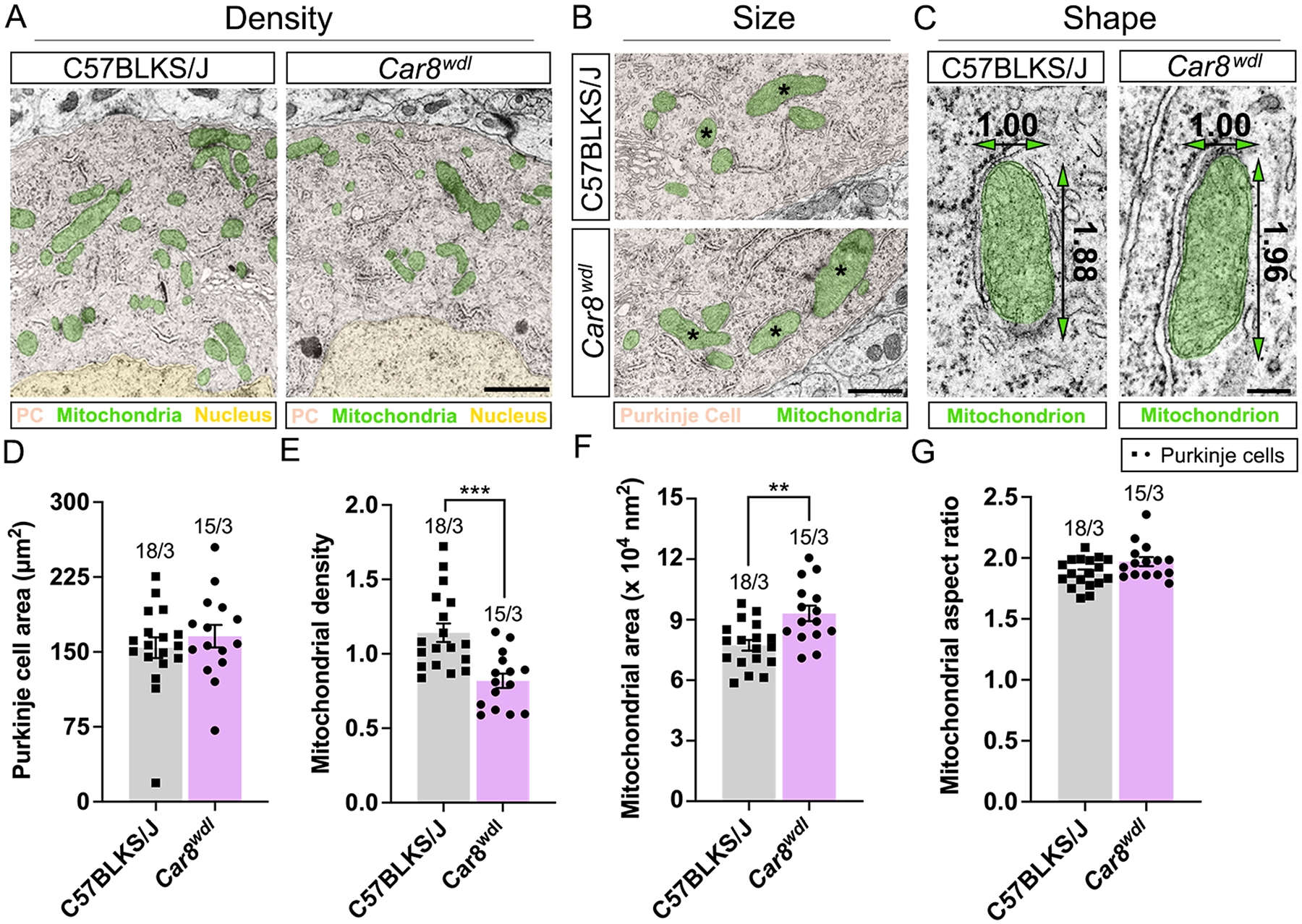
Loss of *Car8* from cerebellar Purkinje cells affects mitochondrial size and density, but not shape. **A.** High-powered (10,000X) TEM images of comparable areas within C57BLKS/J control and *Car8*^*wdl*^ Purkinje cells (pseudo-colored **salmon**) shows fewer mitochondria in the *Car8*^*wdl*^ mutant cerebellum. Mitochondria are pseudo-colored **green** whereas the nuclei are pseudo-colored **yellow**. Scale bar measures 600 nm. **B.** High-powered (10,000X) TEM images showing that *Car8*^*wdl*^ mitochondria are on average larger than C57BLKS/J control mitochondria. Asterisks (*) highlight mitochondria for comparison. Scale bar measures 600 nm. **C.** The predominant shape of mitochondria in C57BLKS/J control and *Car8*^*wdl*^ Purkinje cells is elongated and tubular. Example mitochondria are labeled with their respective dimensions (aspect ratios). Mitochondrial width = minor axis. Mitochondrial length = major axis. Scale bar measures 300 nm. **D-E.** Quantification of cytoplasmic area and mitochondrial density in Purkinje cell soma. **F-G.** Quantification of mitochondrial size and shape. Numbers on bar graphs denote Purkinje cell number (n) / animal number (N), or 15 Purkinje cells from 3 untreated *Car8*^*wdl*^ mice and 18 Purkinje cells from 3 untreated C57BLKS/J control mice. Data analyzed using unpaired two-tailed student’s t-tests after testing for normality. ** p<0.01; *** p<0.001; ns = not significant. Mean ± SEM.

Increased apposition of ER and mitochondria can coincide with changes in mitochondrial density and morphology because of their shared dependence on cell redox state and roles as stress response readouts (Lee et al., 2018; Ahmad et al., 2013; Berridge et al., 2002; Marchi et al., 2014). Mitochondria are elongated, tubular structures that may become fragmented, swollen, or circular when inundated by too much intracellular Ca^2+^ or upon imminent cell death (Ahmad et al., 2013; Wiemerslage et al., 2016; Tan et al., 2011; Kaasik et al., 2007). Given the proposed role of CAR8 in antagonizing IP3R1 receptors, we hypothesized that there would be fewer, but larger, mitochondria in untreated CAR8-deficient Purkinje cells, as observed in cells with increased intracellular Ca^2+^ (Golic et al., 2014). Because we do not observe cell death in *Car8*^*wdl*^ cerebella (White et al., 2016; Miterko et al., 2019), we expect no gross changes in mitochondrial shape. To test these hypotheses, we first measured mitochondrial density in untreated C57BLKS/J control and *Car8*^*wdl*^ Purkinje cell soma. Despite no difference in Purkinje cell soma size (C57BLKS/J control: 154.2 ± 10.4 μm^2^; *Car8*^*wdl*^: 165.7 ± 11.3 μm^2^; p=0.4599), there are significantly fewer mitochondria per area squared in *Car8*^*wdl*^ Purkinje cells (Fig. 2A, D–E; C57BLKS/J control: 1.141 ± 0.062; *Car8*^*wdl*^: 0.819 ± 0.048; p=0.0004). These data suggest the presence of stress within *Car8*^*wdl*^ Purkinje cells, which is corroborated further by increased mitochondrial size in *Car8*^*wdl*^ Purkinje cells (Fig. 2B, F; C57BLKS/J control: 77,318 ± 2,661 nm^2^; *Car8*^*wdl*^: 93,114 ± 3,860 nm^2^; p=0.0016). If mitochondrial shape changes after the loss of *Car8*, then we should observe differences in the aspect ratio of mitochondria. On average, we found no differences between the aspect ratio of C57BLKS/J control and *Car8*^*wdl*^ mitochondria, indicating no significant morphological alterations (Fig. 2C, G). C57BLKS/J control and *Car8*^*wdl*^ mitochondria remain predominantly tubular and elongated, with their lengths almost doubling their widths (C57BLKS/J control: 1.878 ± 0.028; *Car8*^*wdl*^: 1.970 ± 0.038; p=0.0532). Altogether, the data presented in Figs. 1 and 2 show ultrastructural defects that are consistent with Ca^2+^ dysregulation in the untreated cerebellum of CAR8-deficient mice.

### 3.3. Cerebellar DBS rescues mitochondrial density in Car8^wdl^ Purkinje cells

**Fig. 3.**
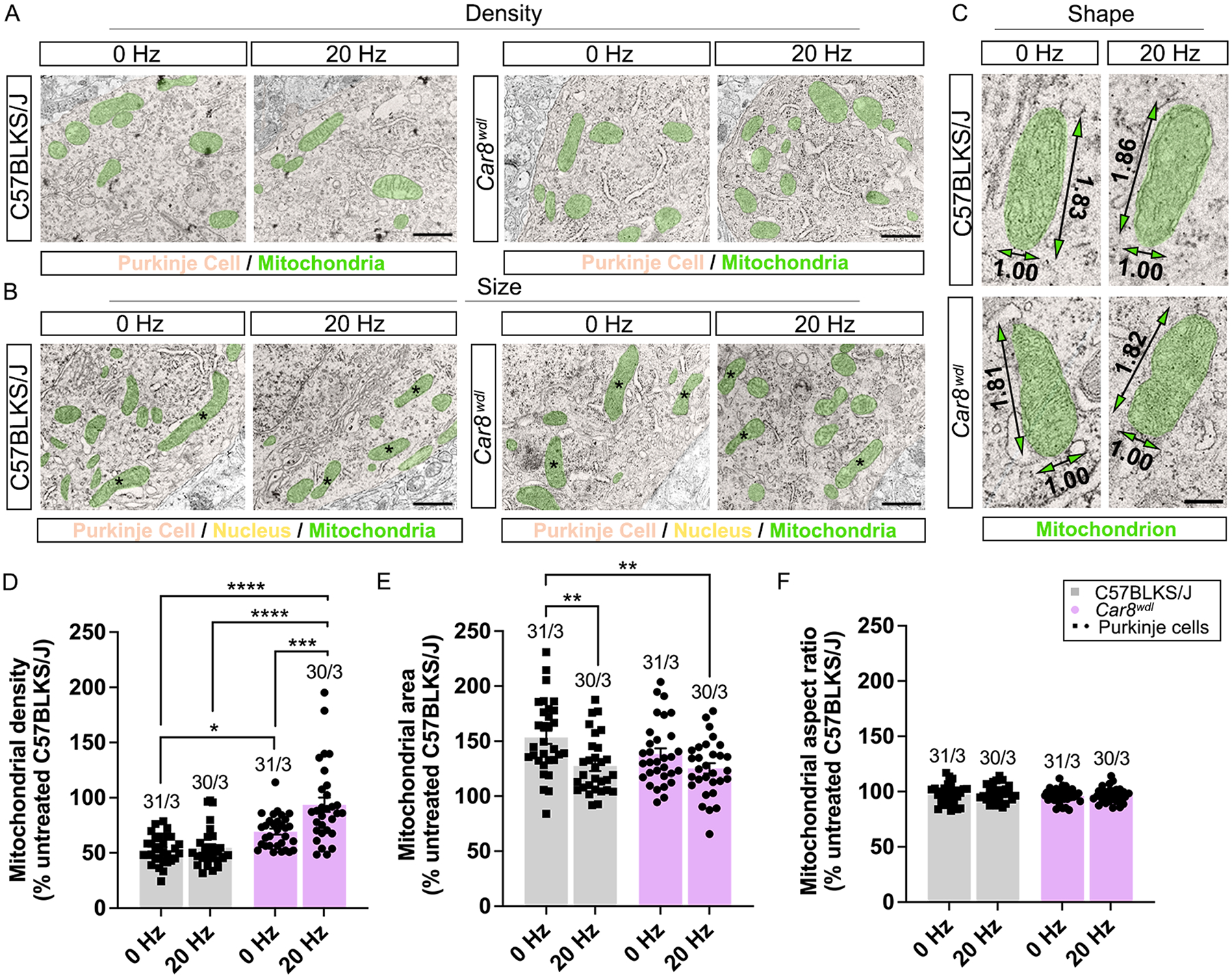
Low-frequency cerebellar DBS normalizes mitochondrial density, but not mitochondrial size in *Car8*^*wdl*^ Purkinje cells. **A.** High-powered (10,000x) TEM images of comparable areas within C57BLKS/J control and *Car8*^*wdl*^ Purkinje cells (pseudo-colored **salmon**) after sham (0 Hz) or DBS (20 Hz) treatments. Mitochondrial density decreases in sham- and DBS-treated C57BLKS/J control Purkinje cells but is unaffected or increased in sham- and DBS-treated *Car8*^*wdl*^ Purkinje cells, respectively. Mitochondria are pseudo-colored **green**. Scale bar measures 600 nm. **B.** High-powered (10,000x) TEM images showing that 20 Hz DBS does not correct *Car8*^*wdl*^ mitochondrial size. Asterisks (*) highlight mitochondria for comparison. Scale bar measures 600 nm. **C.** The predominant shape of mitochondria in sham- or DBS-treated, C57BLKS/J control and *Car8*^*wdl*^ Purkinje cells is elongated and tubular. Example mitochondria are labeled with their respective dimensions (aspect ratios). Mitochondrial width = minor axis. Mitochondrial length = major axis. Scale bar measures 300 nm. **D.** Quantification of mitochondrial density as percentages of untreated C57BLKS/J controls. **E.** Quantification of mitochondrial size as percentages of untreated C57BLKS/J controls. **F.** Quantification of mitochondrial shape as percentages of untreated C57BLKS/J controls. Numbers on bar graphs denote Purkinje cell number (n) / animal number (N). 31 Purkinje cells were analyzed from 3 sham-treated C57BLKS/J control and *Car8*^*wdl*^ mice; 30 Purkinje cells were analyzed from 3 DBS-treated C57BLKS/J control and *Car8*^*wdl*^ mice. Data analyzed using two-way ANOVAs followed by Tukey’s *post hoc* tests. * p<0.05; ** p<0.01; *** p<0.001; **** p<0.0001. Mean ± SEM.

Alterations in mitochondrial size and density are reversible through genetic and pharmacological treatments (Izzo et al., 2017; Dagda et al., 2011; Perdomini et al., 2014), prompting us to ask whether electrical intervention also rescues Purkinje cell nanoarchitecture. In Parkinson’s Disease, STN-DBS normalizes mitochondrial volume and number in neurons of the substantia nigra pars compacta (Mallach et al., 2019). These results show that neurons in circuits targeted by DBS are plastic. To investigate whether cerebellar DBS similarly corrects mitochondrial alterations in Purkinje cells, we quantified mitochondrial density and size in sham and 20 Hz-stimulated C57BLKS/J control and *Car8*^*wdl*^ mice. We found that low-frequency DBS normalizes mitochondrial density in *Car8*^*wdl*^ Purkinje cells to 93.58 ± 5.52 % of untreated C57BLKS/J controls (Fig. 3A, D; n=30 Purkinje cells from 3 mice). Sham treatment does not improve mitochondrial density in *Car8*^*wdl*^ Purkinje cells (Untreated *Car8*^*wdl*^: 71.72 ± 4.22 % of untreated C57BLKS/J controls; 0 Hz: 69.24 ± 2.67 % of untreated C57BLKS/J controls, n=31 Purkinje cells from 3 mice), with numbers remaining significantly lower than that of Purkinje cells in 20 Hz-stimulated *Car8*^*wdl*^ cerebella (Fig. 3A, D; p=0.0002). Sham and low-frequency stimulation treatments half mitochondrial densities in C57BLKS/J control Purkinje cells (Untreated C57BLKS/J: 100.00 ± 5.44 % of untreated C57BLKS/J controls; 0 Hz: 52.88 ± 2.28 % of untreated C57BLKS/J controls, n=31 Purkinje cells from 3 mice; 20 Hz: 54.62 ± 3.22 % control, n=30 Purkinje cells from 3 mice), resulting in phenotypes worse than that of sham and stimulated *Car8*^*wdl*^ Purkinje cells (Fig. 3A, D; 0 Hz C57BLKS/J control vs. 0 Hz *Car8*^*wdl*^, p=0.0217; 0 Hz C57BLKS/J control vs. 20 Hz *Car8*^*wdl*^, p<0.0001; 20 Hz C57BLKS/J control vs. 20 Hz *Car8*^*wdl*^, p<0.0001). Contributing to reduced mitochondrial densities in C57BLKS/J control mice may be larger, ‘swollen’ Purkinje cell soma (Table 1; Untreated: 154.20 ± 10.37 μm^2^; 0 Hz: 281.22 ± 19.50 μm^2^, p=0.0025; 20 Hz: 283.06 ± 23.88 μm^2^, p=0.0023), which is not observed in *Car8*^*wdl*^ cerebella (Table 1; Untreated: 165.70 ± 11.30 μm^2^; 0 Hz: 198.73 ± 16.60 μm^2^, p=0.7792; 20 Hz: 199.91 ± 23.82 μm^2^, p=0.7552). C57BLKS/J control mitochondria proportionately enlarge after surgical intervention, increasing in size to 153.50 ± 6.00 % (0 Hz) and 127.60 ± 4.76 % (20 Hz) of untreated C57BLKS/J controls, now equaling the size of sham and stimulated *Car8*^*wdl*^ mitochondria (Fig. 3B, E; 0 Hz: 138.30 ± 5.13 % of untreated C57BLKS/J controls; 20 Hz: 125.30 ± 4.64 % of untreated C57BLKS/J controls; 0 Hz C57BLKS/J control vs. 0 Hz *Car8*^*wdl*^, p=0.1637; 20 Hz C57BLKS/J control vs. 0 Hz *Car8*^*wdl*^, p=0.4629; 20 Hz C57BLKS/J control vs. 20 Hz *Car8*^*wdl*^, p=0.9896). Mitochondrial shape remains unaffected (Fig. 3C, F; C57BLKS/J control, 0 Hz: 97.44 ± 1.58 % of untreated C57BLKS/J controls; 20 Hz: 98.24 ± 1.36 % of untreated C57BLKS/J controls; *Car8*^*wdl*^: 0 Hz: 96.16 ± 1.21 % of untreated C57BLKS/J controls; 20 Hz: 96.87 ± 1.24 % of untreated C57BLKS/J controls; Genotype, p=0.3299; Frequency, p=0.5784; Genotype x Frequency, p=0.9753). Altogether, our data reveal that intracellular plasticity following neuromodulation is disease- and location-specific, with cerebellar DBS only rescuing mitochondrial density deficits in the ataxia model.

### 3.4. Cerebellar DBS preserves putative mitochondria-ER contacts in Car8^wdl^ Purkinje cells

**Fig. 4.**
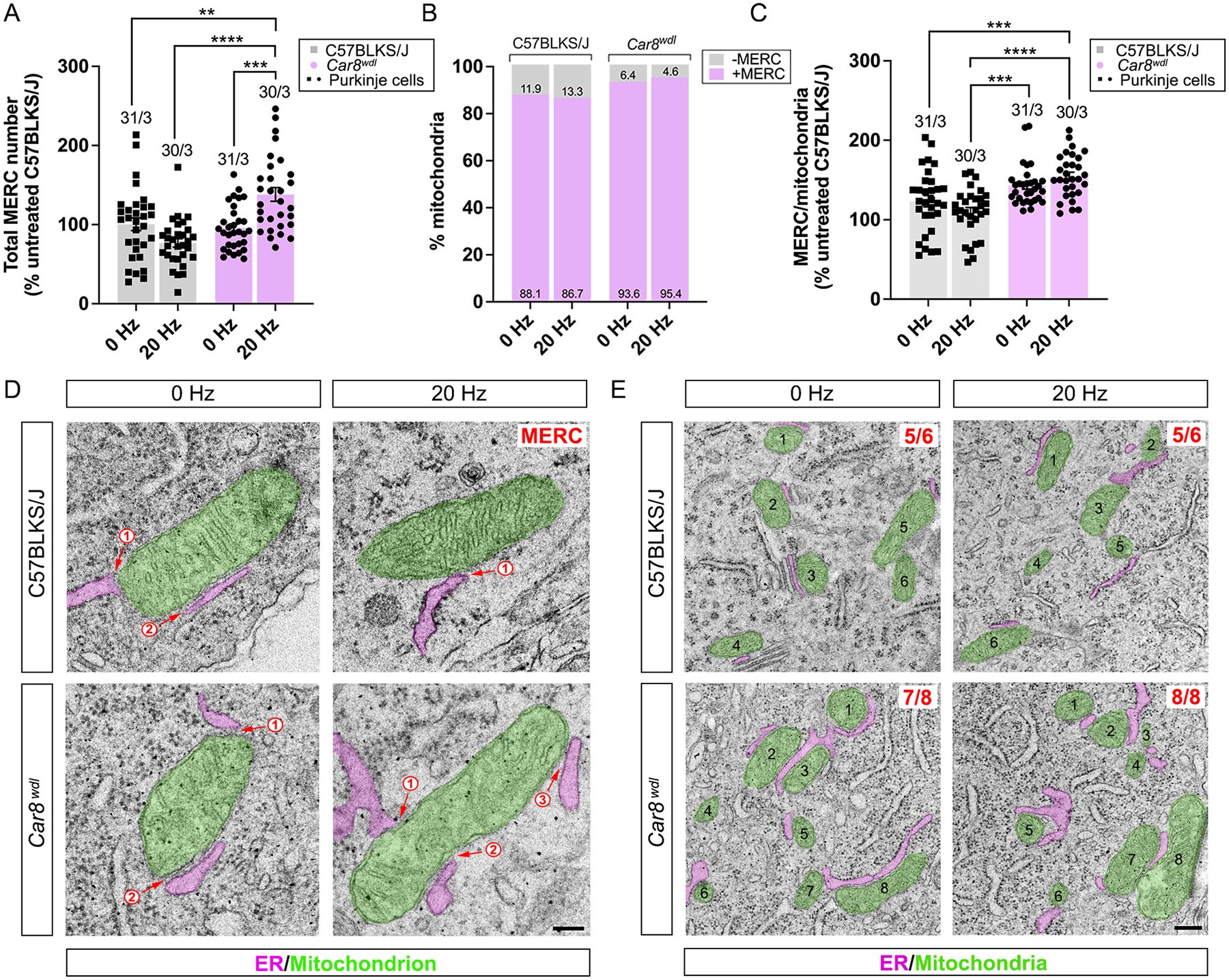
Low-frequency cerebellar DBS preserves mitochondria-ER contact (MERC) frequency and distribution in *Car8*^*wdl*^ Purkinje cells. **A.** Quantification of total MERC number as percentages of untreated C57BLKS/J controls. Stimulated *Car8*^*wdl*^ mice maintain a greater number of MERCs in their Purkinje cells than implanted C57BLKS/J controls and sham *Car8*^*wdl*^ mice. Numbers denote Purkinje cell number (n) / animal number (N), or 31 Purkinje cells from 3 sham mice and 30 Purkinje cells from 3 DBS-treated mice, per genotype. **B.** C57BLKS/J control and *Car8*^*wdl*^ mitochondria subjected to sham and 20 Hz-DBS treatment similarly associate with ER (<30 nm) in Purkinje cells. Numbers on the contingency graph represent the percentage of mitochondria with no MERCs (**gray**) or at least 1 MERC (**magenta**) from 30 to 31 Purkinje cells across 3 C57BLKS/J control sham, 3 C57BLKS/J control stimulated, 3 *Car8*^*wdl*^ sham, and 3 *Car8*^*wdl*^ stimulated mice. **C.** Quantification of MERC distribution across mitochondria, normalized to the mean of untreated C57BLKS/J controls. Stimulated *Car8*^*wdl*^ mice maintain a greater number of MERCs per mitochondrion in their Purkinje cells than sham and stimulated C57BLKS/J control mice. Numbers denote Purkinje cell number (n) / animal number (N), or 31 Purkinje cells from 3 sham mice and 30 Purkinje cells from 3 DBS-treated mice, per genotype. Data analyzed using two-way ANOVAs followed by Tukey’s multiple comparison *post hoc* tests (**A**, **C**) or a chi-squared test (**B**). ** p<0.01; *** p<0.001; **** p<0.0001. Mean ± SEM. **D.** Representative high-powered (10,000x) TEM images of MERC distribution onto mitochondria. Stimulated *Car8*^*wdl*^ mitochondria more often contact ER (**red** numbered arrows) than implanted C57BLKS/J controls and sham *Car8*^*wdl*^ mitochondria. Mitochondria are pseudo-colored **green** whereas ER are pseudo-colored **magenta**. Scale bar measures 150 nm. **E.** The percentage of mitochondria putatively contacting ER (<30 nm) in sham- and DBS-treated *Car8*^*wdl*^ Purkinje cells is not significantly different than that in sham- or DBS-treated C57BLKS/J control Purkinje cells. Mitochondria are numbered and pseudo-colored **green** while ER are pseudo-colored **magenta**. One putative MERC is highlighted per mitochondrion. Numbers on TEM images denote the number of mitochondria associating with ER / total number of mitochondria in the field-of-view. Scale bar measures 300 nm.

Because DBS can improve oxidative stress in neurons (Chen et al., 2024), MERC number may be altered after cerebellar neuromodulation given its role in neutralization responses (Eisner et al., 2018). Quantification of total MERC number reveals increased ER in stimulated, but not sham, *Car8*^*wdl*^ Purkinje cells (Fig. 4A; 0 Hz: 95.67 ± 5.05 % of untreated C57BLKS/J controls; 20 Hz: 138.20 ± 8.63 % of untreated C57BLKS/J controls; 0 Hz C57BLKS/J control vs. 0 Hz *Car8*^*wdl*^, p=0.9611; 0 Hz C57BLKS/J control vs. 20 Hz *Car8*^*wdl*^, p=0.0012). This increase is comparable to what is observed in *Car8*^*wdl*^ mice without surgery (Untreated *Car8*^*wdl*^: 143.50 ± 17.51 % of untreated C57BLKS/J controls, p=0.9988), indicating that cerebellar DBS preserves, rather than further elevates, MERC number in *Car8*^*wdl*^ Purkinje cells. Despite increased ER, sham and 20 Hz-stimulated *Car8*^*wdl*^ mitochondria equally harbor MERCs (0 Hz: 93.59 ± 0.94 % mitochondria; 20 Hz: 95.43 ± 1.37 % mitochondria; p=0.1066) and to similar amounts (0 Hz: 143.00 ± 4.53 % of untreated C57BLKS/J controls; 20 Hz: 154.90 ± 5.01 % of untreated C57BLKS/J controls; p=0.4432), suggesting a greater sequestration of ER around mitochondria in sham *Car8*^*wdl*^ Purkinje cells (Fig. 4B–E). Surgical intervention increases MERC frequency (0 Hz: 88.11 ± 5.23 % mitochondria; 20 Hz: 86.70 ± 2.29 % mitochondria), but not total number or distribution on C57BLKS/J control mitochondria (Total, 0 Hz: 100.50 ± 8.12 % of untreated C57BLKS/J controls, 20 Hz: 76.72 ± 5.43 % of untreated C57BLKS/J controls; Distribution, 0 Hz: 123.40 ± 7.10 % of untreated C57BLKS/J controls, 20 Hz: 109.9 ± 5.50 % of untreated C57BLKS/J controls), relative to *Car8*^*wdl*^ mitochondria (Fig. 4B–E; Frequency, p=0.1066; Total, p<0.0001; Distribution, p<0.0001). When considered alongside data in Fig. 3, electrode implantation, regardless of stimulation paradigm, alters Purkinje cell nanoarchitecture in C57BLKS/J cerebella.

### 3.5. Ultrastructural differences relate to Car8^wdl^ treatment but not efficacy

**Fig. 5.**
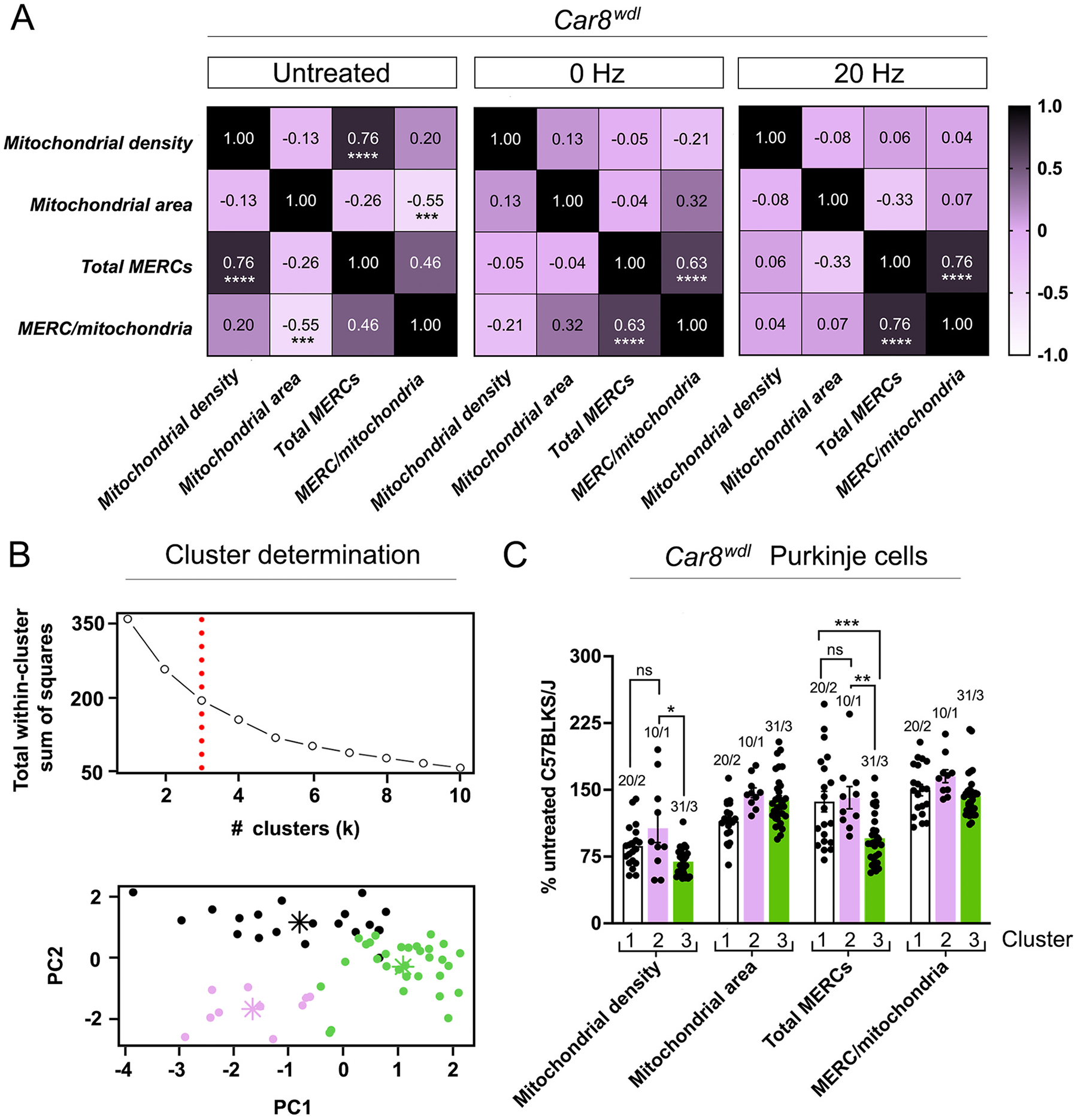
Treatment but not efficacy coincides with mitochondrial density and total MERC number in *Car8*^*wdl*^ Purkinje cells. **A.** Correlation matrices comparing Pearson *r* coefficients across intracellular properties and treatment regimens. *Car8*^*wdl*^ Purkinje cells exhibit similar intracellular compositions after sham and DBS treatments. *** p<0.001; **** p<0.0001. **B.** The k-means algorithm groups *Car8*^*wdl*^ Purkinje cells into 3 clusters, with an elbow plot determining *k* (*k*=3, dotted **red** line). Clusters are visualized using principal components (PC) 1 and 2 and separate colors (Cluster 1 = **black**, Cluster 2 = **magenta**, Cluster 3 = **green**). Each circle represents an individual Purkinje cell and each asterisks represents a cluster mean. **C.** *Car8*^*wdl*^ Purkinje cells are clustered according to treatment, treatment effectiveness, mitochondrial density, and total MERCs. Cluster 1 contains DBS-treated Purkinje cells from *Car8*^*wdl*^ mice that behaviorally improve (97.756 ± 0.317 %, n=20 Purkinje cells from N=2 mice). Cluster 2 contains DBS-treated Purkinje cells from *Car8*^*wdl*^ mice that do not behaviorally improve (-48.153 %, n=10 Purkinje cells from N=1 mouse). Cluster 3 contains sham-treated *Car8*^*wdl*^ Purkinje cells (n=31 Purkinje cells from N=3 mice). Data analyzed using a two-way ANOVA followed by Tukey’s multiple comparison *post hoc* tests. ns = not significant; * p<0.05; ** p<0.01; *** p<0.001. Mean ± SEM.

To determine whether ultrastructural differences in the *Car8*^*wdl*^ Purkinje cells correspond to treatment, correlation matrices were generated, comparing mitochondrial and MERC properties within and between groups. In untreated *Car8*^*wdl*^ mice, Purkinje cell mitochondrial density positively correlates with total MERC number while Purkinje cell mitochondrial area negatively correlates with MERC distribution (Fig. 5A; Density vs. Total MERCs, p=0.001; Area vs. MERC/mitochondria, p=0.032). These significant positive and negative correlations resolve with sham and 20 Hz treatment (Fig. 5A; 0 Hz: Density vs. Total MERCs, p=0.0800; Area vs. MERC/mitochondria, p=0.0706; 20 Hz: Density vs. Total MERCs, p=0.745; Area vs. MERC/mitochondria, p=0.715), indicating that electrode implantation alone alters total and average MERC numbers within *Car8*^*wdl*^ Purkinje cells. Total MERCs and MERC distribution shifts result in positive correlations with one another (Fig. 5A; 0 Hz: Total MERCs vs. MERC/mitochondria, p<0.0001; 20 Hz: Total MERCs vs. MERC/mitochondria, p<0.0001), meaning that sham and stimulated *Car8*^*wdl*^ mitochondria exhibit more putative ER contacts regardless of their size or amount. We conclude from our correlation analyses that mitochondrial and MERC characteristics can differentiate untreated from treated *Car8*^*wdl*^ Purkinje cells but not sham from stimulated *Car8*^*wdl*^ Purkinje cells.

We next performed a k-means cluster analysis to identify similarities in ultrastructural responses among sham and stimulated *Car8*^*wdl*^ Purkinje cells. We found that *Car8*^*wdl*^ Purkinje cells cluster based on differences in treatment (0 Hz vs. 20 Hz), treatment efficacy (−48.153–99.032 % behavioral improvement), mitochondrial density, and total MERC numbers (Fig. 5B–C). For example, 100 % of Purkinje cells in Cluster 3 are from sham-treated *Car8*^*wdl*^ mice and exhibit significantly fewer mitochondria and total MERCs than Purkinje cells in Clusters 1–2, 100 % of which belong to DBS-treated *Car8*^*wdl*^ mice (Fig. 5B–C; Cluster 1 vs. 3: Total MERCs, p=0.0002; Cluster 2 vs. 3: Mitochondrial density, p=0.0284; Total MERCs, p=0.0022). Although Purkinje cells in DBS-treated *Car8*^*wdl*^ mice are further subdivided by DBS efficacy (Cluster 1, 97.756 ± 0.317 % behavioral improvement; Cluster 2: − 48.153 % behavioral improvement), no significant deviations from increased mitochondrial density or total MERCs are observed (Fig. 5C; Cluster 1 vs. 2: Mitochondrial density, p=0.8434; Total MERCs, p>0.9999). Our data support the hypothesis that low-frequency cerebellar DBS efficacy relates, in part, to improved mitochondrial density and preserved putative mitochondrial-ER contacts. Other factors, such as altered synaptic activity, likely contributes to distinguishing mice that favorably respond to 20 Hz DBS from those that do not. Understanding specifically how ultrastructural differences in the Purkinje cells mediate behavioral and synaptic changes remains to be elucidated.

## 4. Discussion

DBS is a promising therapy for cerebellar ataxia (Miterko et al., 2021; Teixeira et al., 2015; Cury et al., 2019; Anderson et al., 2019). Our prior characterization of DBS for cerebellar ataxia in *Car8*^*wdl*^ mice revealed long-lasting motor benefits, reliant on low, beta-frequencies and Purkinje cell neurotransmission (Miterko et al., 2021). Whether intracellular changes within Purkinje cells underlie the persistent benefits of cerebellar DBS is unknown. Here, we show that low-frequency stimulation of *Car8*^*wdl*^ cerebella alters the intracellular organization of Purkinje cells by increasing mitochondrial density and preserving MERC distribution. *Car8*^*wdl*^ mice model human congenital ataxia, where a predisposition to abnormal quadrupedal gait is hypothesized to arise from Ca^2+^ dysregulation, a consequence of reduced CA8 protein in the cerebellum (CAR8, rodents) (Türkmen et al., 2009). CAR8 is an allosteric inhibitor of IP3R1, an ER-bound receptor that transfers Ca^2+^ to the cytosol or mitochondria at contact sites (Hirota et al., 2003; Ahumada-Castro et al., 2021). In cells with high intracellular Ca^2+^, more MERCs form (Lee et al., 2018), consistent with our observations in Purkinje cells of untreated *Car8*^*wdl*^ mice (Fig. 1). Increased MERCs may offer neuroprotection through maintaining cellular metabolic rates and promoting mitophagy or mitochondrial remodeling (Garrido-Maraver et al., 2020; Casas-Martinez et al., 2025; Bassot et al., 2023), as seen early in neurological disease (Peng et al., 2025). *Car8*^*wdl*^ Purkinje cells do not degenerate and exhibit fewer mitochondria, supporting mitophagy occurring (Fig. 2) (White et al., 2016). Another possibility is that *Car8*^*wdl*^ mitochondria fuse to accommodate higher intracellular Ca^2+^ levels and improve metabolism, resulting in fewer but larger mitochondria (Figs. 1–2) (Eisner et al., 2018; Kowaltowski et al., 2019; Leung et al., 2021; Kann and Kovács, 2007; Jenkins et al., 2024).

Not only does mitochondrial number and size change in response to endogenous cues such as intracellular Ca^2+^ levels (Golic et al., 2014), but they also change after exogenous stimuli, including electrical stimulation (Reichmann et al., 1985; Schils et al., 2015). In cultured neuroblastoma cells, electrical stimulation promotes survival through increasing mitochondrial fusion (Love et al., 2019). *In vivo* stimulation of the cerebellar fastigial nuclei restores cellular respiration and suppresses apoptosis by increasing mitochondrial Ca^2+^ uptake capacity (Zhou et al., 2005). *Post-mortem* studies on Parkinson’s disease brains show that mitochondria in diseased neural circuits equally respond to DBS protocols, with mitochondria in substantia nigra pars compacta neurons increasing in volume and number after STN-DBS (Mallach et al., 2019). We similarly found increased mitochondrial numbers in stimulated *Car8*^*wdl*^ cerebella (Fig. 3), indicating that DBS enhances mitochondrial biogenesis or fission in Purkinje cells. Normalization of mitochondrial density, but not size, to untreated C57BLKS/J control levels reveals incomplete repair, likely due to constituent CAR8 loss which continues to impair IP3R1 gating (Hirota et al., 2003). Electrical stimulation may also raise intracellular Ca^2+^ (Khatib et al., 2004; Adams et al., 2017), contributing to its sustained elevation in *Car8*^*wdl*^ Purkinje cells. Considering this, the combination of more and larger mitochondria in stimulated *Car8*^*wdl*^ Purkinje cells suggests an adaptive mechanism to increase cell metabolic rates.

Preserved increases in MERC frequency and distribution in *Car8*^*wdl*^ Purkinje cells after low-frequency cerebellar DBS support conclusions of an adaptive response (Fig. 4). Neuronal swelling in C57BLKS/J control cerebella after surgical intervention suggests crosstalk with mitochondrial respiration pathways (Patel et al., 1998). MERCs support mitochondrial respiration, with increased numbers increasing energy production in diseased neurons (Leung et al., 2021; Sathyamurthy et al., 2024). Energy is required by neurons to control cell excitability, and in cerebellar Purkinje cells, production is large to maintain their tonic firing activity (Howarth et al., 2010; Biser et al., 2000). Transient influxes of Ca^2+^ following Purkinje cell activation increases ion transport demand for adenosine triphosphate (ATP), which drive physiological responses through sodium potassium pumps and Ca^2+^-activated potassium channels (Kann and Kovács, 2007; Forrest et al., 2012; Erecińska and Dagani, 1990; Ivannikov et al., 2010). ATP production fluctuations affect Purkinje cell firing, with increases enhancing and decreases suppressing synaptic activity (Deitmer et al., 2006; Casel et al., 2005; Hamann et al., 2005). Increased MERCs, combined with larger and more mitochondria, may represent attempts at sustaining *Car8*^*wdl*^ Purkinje cell excitability. Aside from generating more ATP, MERCs support exocytosis (Dentoni et al., 2022). *Car8*^*wdl*^ Purkinje cells fire in bursts, with more pausing, which are hallmarks of impaired Ca^2+^ clearance and increased exocytosis (White et al., 2016; Montefusco and Pedersen, 2019; De Schutter and Steuber, 2009).

Analyses were performed on 2-dimensional TEM images of post-fixed tissue given our interests in studying mitochondrial morphology and MERCs after cerebellar stimulation. Only TEM allows for simultaneous measurements of bulk mitochondrial properties and MERC contact distance in intact cells and in the absence of exogenous chemical reactions (Giamogante et al., 2020; Neikirk et al., 2023). Purkinje cells also contain more MERCs than the average cell type (Fecher et al., 2019), making TEM a more attractive option than fluorescent probe- or immunodetection based approaches (Giamogante et al., 2020). However, this limits our investigation into DBS effects on Purkinje cell biochemistry. It will be important to confirm our ultrastructural analyses with biochemical assays and bridge our anatomical findings with that of cell physiology and animal behavior, especially after determining that intracellular composition coincides with DBS treatment (Fig. 5). If cerebellar neuromodulation impacts Purkinje cell metabolism, as studies suggest (Cui et al., 2023; Brusa et al., 2012), then this could advance our understanding of DBS for neurodegenerative ataxias. For example, Purkinje cell firing irregularities, aberrant Ca^2+^ signaling, and altered mitochondrial properties are found in *Car8*^*wdl*^ cerebella and neurodegenerative ataxias (White et al., 2016; Miterko et al., 2019; Hirota et al., 2003; Girard et al., 2012; Zhu et al., 2024; Leung et al., 2024; Maltecca et al., 2015; Rodríguez et al., 2020; Yeo et al., 2021; Shimobayashi and Kapfhammer, 2018; Meera et al., 2016). Currently, there are variable reports on neuromodulation efficacy for neurodegenerative ataxias, with consistent benefits observed when the cerebellum is stimulated as a part of multi-target approaches (Cui et al., 2023; Benussi et al., 2021) or combined with rehabilitative training (Brito et al., 2024a, 2024b; Portaro et al., 2019), but not when ataxia is mild (Maas et al., 2022; Benussi et al., 2017; Shi et al., 2023) or a co-morbidity (Teixeira et al., 2015; Cury et al., 2022, 2015). Because different cerebellar disorders exhibit different neuronal signatures (van der Heijden et al., 2024), it is possible that the stimulation paradigms employed were not targeting ataxia-specific circuit changes when ataxia presents alongside other symptoms. Additionally, neurodegeneration may interfere with stimulation results. Case reports of patients with neurodegenerative ataxias support this by showing little-to-no improvement in gait after cerebellar stimulation when cell loss is severe and residual Purkinje cell functioning is limited (John et al., 2017; Grimaldi and Manto, 2013) but greater improvements in gait when Purkinje cell connectivity remains intact (Benussi et al., 2017; Farzan et al., 2013; Naeije et al., 2023). The degree to which Purkinje cell connectivity is preserved depends on age and intracerebellar location, with older Purkinje cells exhibiting more mitochondrial dysfunction than younger (Zhang et al., 2010) and posterior Purkinje cells exhibiting more resistance to degeneration than anterior (Torvik et al., 1986; Andersen et al., 2003). Purkinje cell vulnerability is further compartmentalized, with cerebellar degeneration occurring in zebrin II-negative parasagittal stripes, corresponding to areas of higher intrinsic excitability and with lower amounts of neuroprotective proteins (Donofrio et al., 2025; Zhou et al., 2014; Xiao et al., 2014; Armstrong et al., 2011). Together, these data suggest that Purkinje cell health and residual activity determine stimulation effectiveness in ataxia. When Purkinje cell functions are too compromised, electric or aerobic stimulation of downstream connections may help bypass the damage so that benefits could still manifest. Indeed, finding that DBS efficacy depends on more than ultrastructural composition supports this hypothesis (Fig. 5).

Our prior characterization of cerebellar DBS effectiveness in ataxia also show that younger Purkinje cells and intact neurotransmission are required for behavioral improvements (Miterko et al., 2021). Purkinje cells provide the sole output of the cerebellar cortex, serving as an integrative hub for sensory and motor information, emanating from the cerebral cortex and spinal cord. Its intrinsic pace-making activity contributes to the transmission of this information onto cerebellar nuclei, which then establish and maintain motor control. Purkinje cell pace-making is highly dependent on intracellular Ca^2+^ homeostasis and mitochondrial respiration (Egorova et al., 2015; Stefely et al., 2016), with precision decreasing if Ca^2+^ signaling and ATP production decreases (Walter et al., 2006; Hamann et al., 2005). Restoration of intracellular Ca^2+^ homeostasis and mitochondrial functions rescues Purkinje cell pace-making, prevents degeneration, and improves motor behavior (Manolaras et al., 2023; Maltecca et al., 2015; Ferro et al., 2017; Stucki et al., 2016). Whether the mechanism-of-action for cerebellar DBS involves restoring Purkinje cell pace-making as some pharmaceuticals do—1-ethyl-2-benzimidazolinone (EBIO), chlorzoxazone, and 4-aminopyridine (4-AP)—is unclear (Walter et al., 2006; White et al., 2016; Alviña and Khodakhah, 2010). Electrical stimulation can modify Ca^2+^ and ATP-coupled ion channel activity (Huang and Shakkottai, 2023; Karatum et al., 2023), which, in Purkinje cells, can improve time interval learning, then motor coordination through generating specific spatiotemporal patterns (Majoral et al., 2020).

Besides phenocopying medications by functionally converging on ion channels, cerebellar DBS may enhance exercise-induced motor recovery, as does transcranial magnetic stimulation (TMS) after spinal cord injury (Jo and Perez, 2020). We previously found that *Car8*^*wdl*^ mice only improve if cerebellar DBS is paired with exercise training (Miterko et al., 2021). Here, we demonstrated that low-frequency cerebellar DBS, when paired with rotarod exercise, modulates Purkinje cell mitochondrial density (Fig. 3). Exercise training specifically increases mitochondrial biogenesis in rodent cerebella (Steiner et al., 2011), supporting the possibility of DBS preserving exercise-mediated intracellular plasticity gains. Although MERC number and distribution have not been quantified in the cerebellum after exercise, enhanced Ca^2+^ retention has been observed, as well as remodeled mitochondria-associated membranes in skeletal muscle (Lee et al., 2021; Picard et al., 2013; Li et al., 2024), suggesting that training could alter cerebellar MERC properties. Future studies involving live cell tracking after stimulation and combinatorial, functional experiments would resolve whether cerebellar neuromodulation alters mitochondrial and Ca^2+^ dynamics as well as reveal ionic mechanisms and whether DBS efficacy could be expanded beyond previously defined therapeutic windows (Miterko et al., 2021).

Understanding the cellular changes spanning the progression and treatment of neurological diseases will be invaluable towards tailoring therapeutic strategies. Our findings of intracellular plasticity after electrical stimulation in an ataxia mouse model suggest that functional recovery of the cerebellum may depend on a combination of factors, including increased Purkinje cell bioenergetics, increased intracellular calcium control, and improved cerebello-thalamo-cortical communication. What this may mean for the clinic is individually thresholding DBS parameters or pairing electrical stimulation with rehabilitative exercises and medications that promote mitochondrial respiration and intracellular calcium clearance in cerebellar Purkinje cells. Improving cellular metabolism likely needs to occur in conjunction with alterations to synaptic communication in order to optimize and prolong patient benefits. Stimulating the nucleus accumbens demonstrates this, where high-frequency DBS alleviates depressive symptoms by simultaneously increasing mitochondrial function and dopamine release (Kim et al., 2016; Li et al., 2023; Dijk et al., 2012).

The extent to which bioenergetic changes sustain synaptic activity changes may not depend on DBS frequency as much as on DBS targeting or underlying pathology. For example, stimulating the STN at the same frequency as the nucleus accumbens (130 Hz), restores mitochondrial size and number within neurons of the substantia nigra pars compacta but not mitochondrial distance from presynaptic dopaminergic terminals or dopamine release (Mallach et al., 2019; Strafella et al., 2003). Increased antioxidant activity accompanies mitochondrial alterations but does not correlate with motor gains in Parkinson’s disease patients (Chen et al., 2024), suggesting that STN-DBS does not improve cellular health to directly improve behavior via dopaminergic output. Instead, STN-DBS mimics the neuromodulatory effects of dopamine by suppressing pathological beta-band oscillations between motor regions rather than restoring synaptic connections (McConnell et al., 2012; Binns et al., 2025). A similar phenomenon is observed in dystonia and tremor, where high-frequency DBS disrupts pathological communication to reduce rigidity and oscillatory behaviors (Piña-Fuentes et al., 2020; Whitmer et al., 2013; Barow et al., 2014; Brown et al., 2020; Yousif et al., 2017). Taken into consideration alongside our findings, DBS may similarly alter intracellular organization at high or low stimulation frequencies. Whether the intracellular changes achieved with low frequency DBS directly impacts neurotransmission remains to be elucidated but will be important for understanding long-term efficacy and translatability across stimulation paradigms.

## Figures and Tables

**Table 1:** A comprehensive overview of the data compared and statistical tests used in this study.

Comparison	Normalization	Statistical Test	*n* (Purkinje Cells)	*N* (mice)	Mean(s) ± SEM	Raw Mean(s) ± SEM	Probability	Significant?	Figure
Purkinje cell number intra- and inter-variability	None	Two-tailed, paired student’s t-test	18 C57BLKS/J	3 C57BLKS/J	17.11 ± 6.13 mitochondria (intra-variability) vs. 22.80 ± 2.63 mitochondria (inter-variability)	No normalization performed	p=0.2479	No	-
Purkinje cell size intra- and inter-variability	None	Two-tailed, paired student’s t-test	18 C57BLKS/J	3 C57BLKS/J	13.01 ± 2.53 mitochondria (intra-variability) vs. 17.85 ± 0.34 mitochondria (inter-variability)	No normalization performed	p=0.2337	No	-
Purkinje cell putative ER contacts intra- and inter-variability	None	Two-tailed, paired student’s t-test	18 C57BLKS/J	3 C57BLKS/J	12.91 ± 2.68 mitochondria (intra-variability) vs. 20.75 ± 2.34 mitochondria (inter-variability)	No normalization performed	p=0.2527	No	-
% Mitochondria with MERCs	None	Chi-Squared Test	18 C57BLKS/J, 15 *Car8*^*wdl*^	3 C57BLKS/J, 3 *Car8*^*wdl*^	77.1 ± 8.3% (C57BLKS/J) vs. 92.4 ± 0.2% (*Car8*^*wdl*^)	No normalization performed	p=0.0060	Yes	1C
Total MERC number	None	Two-tailed, unpaired student’s t-test	18 C57BLKS/J, 15 *Car8*^*wdl*^	3 C57BLKS/J, 3 *Car8*^*wdl*^	153.1 ± 16.3 MERCs (C57BLKS/J) vs. 219.7 ± 26.8 MERCs (*Car8*^*wdl*^)	No normalization performed	p=0.0352	Yes	1D
MERC/mitochondria	None	Two-tailed, unpaired student’s t-test	18 C57BLKS/J, 15 *Car8*^*wdl*^	3 C57BLKS/J, 3 *Car8*^*wdl*^	1.74 ± 0.04 MERC/mito (C57BLKS/J) vs. 2.00 ± 0.04 MERC/mito (*Car8*^*wdl*^)	No normalization performed	p<0.0001	Yes	1E
Purkinje cell area	None	Two-tailed, unpaired student’s t-test	18 C57BLKS/J, 15 *Car8*^*wdl*^	3 C57BLKS/J, 3 *Car8*^*wdl*^	154.20 ± 10.37 μm^2^ (C57BLKS/J) vs. 165.70 ± 11.30 μm^2^ (*Car8*^*wdl*^)	No normalization performed	p=0.4599	No	2D
Mitochondrial density	None	Two-tailed, unpaired student’s t-test	18 C57BLKS/J, 15 *Car8*^*wdl*^	3 C57BLKS/J, 3 *Car8*^*wdl*^	1.14 ± 0.06 mito/PC (C57BLKS/J) vs. 0.82 ± 0.05 mito/PC (*Car8*^*wdl*^)	No normalization performed	p=0.0004	Yes	2E
Mitochondrial area	None	Two-tailed, unpaired student’s t-test	18 C57BLKS/J, 15 *Car8*^*wdl*^	3 C57BLKS/J, 3 *Car8*^*wdl*^	77,318 ± 2,661 μm^2^ (C57BLKS/J) vs. 93,114 ± 3,860 μm^2^ (*Car8*^*wdl*^)	No normalization performed	p=0.0016	Yes	2F
Mitochondrial aspect ratio	None	Two-tailed, unpaired student’s t-test	18 C57BLKS/J, 15 *Car8*^*wdl*^	3 C57BLKS/J, 3 *Car8*^*wdl*^	1.878 ± 0.028 μm^2^ (C57BLKS/J) vs. 1.970 ± 0.038 μm^2^ (*Car8*^*wdl*^)	No normalization performed	p=0.0532	No	2G
Mitochondrial density	Yes; % Untreated C57BLKS/J Controls	Two-way ANOVA	31 C57BLKS/J (0 Hz), 31 *Car8*^*wdl*^ (0 Hz); 30 C57BLKS/J (20 Hz), 30 *Car8*^*wdl*^ (20 Hz)	3 C57BLKS/J (0 Hz), 3 *Car8*^*wdl*^ (0 Hz); 3 C57BLKS/J (20 Hz), 3 *Car8*^*wdl*^ (20 Hz)	1.878 ± 0.028 μm^2^ (C57BLKS/J) vs. 1.970 ± 0.038 μm^2^ (*Car8*^*wdl*^)	0.603 ± 0.026 mito/PC (C57BLKS/J, 0 Hz) vs. 0.623 ± 0.037 mito/PC (C57BLKS/J, 20 Hz) vs. 0.790 ± 0.030 mito/PC (*Car8*^*wdl*^, 0 Hz) vs. 1.068 ± 0.074 mito/PC (*Car8*^*wdl*^, 20 Hz)	Genotype, p<0.0001; Treatment, p=0.0014; Genotype x Treatment, p=0.0055	Yes	3D
		Tukey’s multiple comparisons test	31 C57BLKS/J (0 Hz) vs. 30 C57BLKS/J (20 Hz)	3 C57BLKS/J (0 Hz) vs. 3 C57BLKS/J (20 Hz)	52.88 ± 2.28% (C57BLKS/J, 0 Hz) vs. 54.62 ± 3.22% (C57BLKS/J, 20 Hz)	0.603 ± 0.026 mito/PC (C57BLKS/J, 0 Hz) vs. 0.623 ± 0.037 mito/PC (C57BLKS/J, 20 Hz)	p=0.9899	No	3D
		Tukey’s multiple comparisons test	31 C57BLKS/J (0 Hz) vs. 31 *Car8*^*wdl*^ (0 Hz)	3 C57BLKS/J (0 Hz) vs. 3 *Car8*^*wdl*^ (0 Hz)	52.88 ± 2.28% (C57BLKS/J, 0 Hz) vs. 69.24 ± 2.67% (*Car8*^*wdl*^, 0 Hz)	0.603 ± 0.026 mito/PC (C57BLKS/J, 0 Hz) vs. 0.790 ± 0.030 mito/PC (*Car8*^*wdl*^, 0 Hz)	p=0.0217	Yes	3D
		Tukey’s multiple comparisons test	31 C57BLKS/J (0 Hz) vs. 30 *Car8*^*wdl*^ (20 Hz)	3 C57BLKS/J (0 Hz) vs. 3 *Car8*^*wdl*^ (20 Hz)	52.88 ± 2.28% (C57BLKS/J, 0 Hz) vs. 93.58 ± 6.52% (*Car8*^*wdl*^, 20 Hz)	0.603 ± 0.026 mito/PC (C57BLKS/J, 0 Hz) vs. 1.068 ± 0.074 mito/PC (*Car8*^*wdl*^, 20 Hz)	p<0.0001	Yes	3D
		Tukey’s multiple comparisons test	31 *Car8*^*wdl*^ (0 Hz) vs. 30 C57BLKS/J (20 Hz)	3 *Car8*^*wdl*^ (0 Hz) vs. 3 C57BLKS/J (20 Hz)	54.62 ± 3.22% (C57BLKS/J, 20 Hz) vs. 69.24 ± 2.67% (*Car8*^*wdl*^, 0 Hz)	0.623 ± 0.037 mito/PC (C57BLKS/J, 20 Hz) vs. 0.790 ± 0.030 mito/PC (*Car8*^*wdl*^, 0 Hz)	p=0.0525	No	3D
		Tukey’s multiple comparisons test	30 C57BLKS/J (20 Hz) vs. 30 *Car8*^*wdl*^ (20 Hz)	3 C57BLKS/J (20 Hz) vs. 3 *Car8*^*wdl*^ (20 Hz)	54.62 ± 3.22% (C57BLKS/J, 20 Hz) vs. 93.58 ± 6.52% (*Car8*^*wdl*^, 20 Hz)	0.623 ± 0.037 mito/PC (C57BLKS/J, 20 Hz) vs. 1.068 ± 0.074 mito/PC (*Car8*^*wdl*^, 20 Hz)	p<0.0001	Yes	3D
		Tukey’s multiple comparisons test	31 *Car8*^*wdl*^ (0 Hz) vs. 30 *Car8*^*wdl*^ (20 Hz)	3 *Car8*^*wdl*^ (0 Hz) vs. 3 *Car8*^*wdl*^ (20 Hz)	69.24 ± 2.67% (*Car8*^*wdl*^, 0 Hz) vs. 93.58 ± 6.52% (*Car8*^*wdl*^, 20 Hz)	0.790 ± 0.030 mito/PC (*Car8*^*wdl*^, 0 Hz) vs. 1.068 ± 0.074 mito/PC (*Car8*^*wdl*^, 20 Hz)	p=0.0002	Yes	3D
Purkinje cell area	None	Two-Way ANOVA	18 C57BLKS/J (No Surgery), 15 *Car8*^*wdl*^ (No Surgery); 31 C57BLKS/J (0 Hz), 31 *Car8*^*wdl*^ (0 Hz); 30 C57BLKS/J (20 Hz), 30 *Car8*^*wdl*^ (20 Hz)	3 C57BLKS/J (No Surgery), 3 *Car8*^*wdl*^ (No Surgery); 3 C57BLKS/J (0 Hz), 3 *Car8*^*wdl*^ (0 Hz); 3 C57BLKS/J (20 Hz), 3 *Car8*^*wdl*^ (20 Hz)	149.132 ± 11.456 μm^2^ (C57BLKS/J, No Surgery) vs. 281.225 ± 19.504 μm^2^ (C57BLKS/J, 0 Hz) vs. 283.063 ± 23.881 μm^2^ (C57BLKS/J, 20 Hz) vs. 165.368 ± 6.834 μm^2^ (*Car8*^*wdl*^, No Surgery) vs. 198.728 ± 16.604 μm^2^ (*Car8*^*wdl*^, 0 Hz) vs. 199.908 ± 23.817 μm^2^ (*Car8*^*wdl*^, 20 Hz)	No normalization performed	Genotype, p=0.0056; Treatment, p=0.0007; Genotype x Treatment, p=0.0267	Yes	-
		Tukey’s multiple comparisons test	18 C57BLKS/J (No Surgery) vs. 31 C57BLKS/J (0 Hz)	3 C57BLKS/J (No Surgery) vs. 3 C57BLKS/J (0 Hz)	149.132 ± 11.456 μm^2^ (C57BLKS/J, No Surgery) vs. 281.225 ± 19.504 μm^2^ (C57BLKS/J, 0 Hz)	No normalization performed	p=0.0025	Yes	-
		Tukey’s multiple comparisons test	18 C57BLKS/J (No Surgery) vs. 30 C57BLKS/J (20 Hz)	3 C57BLKS/J (No Surgery) vs. 3 C57BLKS/J (20 Hz)	149.132 ± 11.456 μm^2^ (C57BLKS/J, No Surgery) vs. 283.063 ± 23.881 μm^2^ (C57BLKS/J, 20 Hz)	No normalization performed	p=0.0023	Yes	-
		Tukey’s multiple comparisons test	15 *Car8*^*wdl*^ (No Surgery) vs. 31 *Car8*^*wdl*^ (0 Hz)	3 *Car8*^*wdl*^ (No Surgery) vs. 3 *Car8*^*wdl*^ (0 Hz)	165.368 ± 6.834 μm^2^ (*Car8*^*wdl*^, No Surgery) vs. 198.728 ± 16.604 μm^2^ (*Car8*^*wdl*^, 0 Hz)	No normalization performed	p=0.7792	No	-
		Tukey’s multiple comparisons test	15 *Car8*^*wdl*^ (No Surgery) vs. 30 *Car8*^*wdl*^ (20 Hz)	3 *Car8*^*wdl*^ (No Surgery) vs. 3 *Car8*^*wdl*^ (20 Hz)	165.368 ± 6.834 μm^2^ (*Car8*^*wdl*^, No Surgery) vs. 199.908 ± 23.817 μm^2^ (*Car8*^*wdl*^, 20 Hz)	No normalization performed	p=0.7552	No	-
Mitochondrial area	Yes; % Untreated C57BLKS/J Controls	Two-way ANOVA	31 C57BLKS/J (0 Hz), 31 *Car8*^*wdl*^ (0 Hz); 30 C57BLKS/J (20 Hz), 30 *Car8*^*wdl*^ (20 Hz)	3 C57BLKS/J (0 Hz), 3 *Car8*^*wdl*^ (0 Hz); 3 C57BLKS/J (20 Hz), 3 *Car8*^*wdl*^ (20 Hz)	153.5 ± 6.0% (C57BLKS/J, 0 Hz) vs. 127.6 ± 4.8% (C57BLKS/J, 20 Hz) vs. 138.3 ± 5.1% (*Car8*^*wdl*^, 0 Hz) vs. 125.3 ± 4.6% (*Car8*^*wdl*^, 20 Hz)	118,656 ± 4,639 μm^2^ (C57BLKS/J, 0 Hz) vs. 98,657 ± 3,682 μm^2^ (C57BLKS/J, 20 Hz) vs. 106,942 ± 3,969 μm^2^ (*Car8*^*wdl*^, 0 Hz) vs. 96,886 ± 3,590 μm^2^ (*Car8*^*wdl*^, 20 Hz)	Genotype, p=0.0948; Treatment, p=0.0003; Genotype x Treatment, p=0.2168	Yes/No	3E
		Tukey’s multiple comparisons test	31 C57BLKS/J (0 Hz), 31 *Car8*^*wdl*^ (0 Hz); 30 C57BLKS/J (20 Hz), 30 *Car8*^*wdl*^ (20 Hz)	3 C57BLKS/J (0 Hz) vs. 3 C57BLKS/J (20 Hz)	153.5 ± 6.0% (C57BLKS/J, 0 Hz) vs. 127.6 ± 4.8% (C57BLKS/J, 20 Hz)	118,656 ± 4,639 μm^2^ (C57BLKS/J, 0 Hz) vs. 98,657 ± 3,682 μm^2^ (C57BLKS/J, 20 Hz)	p=0.0033	Yes	3E
		Tukey’s multiple comparisons test	31 C57BLKS/J (0 Hz), 31 *Car8*^*wdl*^ (0 Hz); 30 C57BLKS/J (20 Hz), 30 *Car8*^*wdl*^ (20 Hz)	3 C57BLKS/J (0 Hz) vs. 3 *Car8*^*wdl*^ (0 Hz)	153.5 ± 6.0% (C57BLKS/J, 0 Hz) vs. 138.3 ± 5.1% (*Car8*^*wdl*^, 0 Hz)	118,656 ± 4,639 μm^2^ (C57BLKS/J, 0 Hz) vs. 106,942 ± 3,969 μm^2^ (*Car8*^*wdl*^, 0 Hz)	p=0.1637	No	3E
		Tukey’s multiple comparisons test	31 C57BLKS/J (0 Hz), 31 *Car8*^*wdl*^ (0 Hz); 30 C57BLKS/J (20 Hz), 30 *Car8*^*wdl*^ (20 Hz)	3 C57BLKS/J (0 Hz) vs. 3 *Car8*^*wdl*^ (20 Hz)	153.5 ± 6.0% (C57BLKS/J, 0 Hz) vs. 125.3 ± 4.6% (*Car8*^*wdl*^, 20 Hz)	118,656 ± 4,639 μm^2^ (C57BLKS/J, 0 Hz) vs. 96,886 ± 3,590 μm^2^ (*Car8*^*wdl*^, 20 Hz)	p=0.0011	Yes	3E
		Tukey’s multiple comparisons test	31 C57BLKS/J (0 Hz), 31 *Car8*^*wdl*^ (0 Hz); 30 C57BLKS/J (20 Hz), 30 *Car8*^*wdl*^ (20 Hz)	3 *Car8*^*wdl*^ (0 Hz) vs. 3 C57BLKS/J (20 Hz)	127.6 ± 4.8% (C57BLKS/J, 20 Hz) vs. 138.3 ± 5.1% (*Car8*^*wdl*^, 0 Hz)	98,657 ± 3,682 μm^2^ (C57BLKS/J, 20 Hz) vs. 106,942 ± 3,969 μm^2^ (*Car8*^*wdl*^, 0 Hz)	p=0.4629	No	3E
		Tukey’s multiple comparisons test	31 C57BLKS/J (0 Hz), 31 *Car8*^*wdl*^ (0 Hz); 30 C57BLKS/J (20 Hz), 30 *Car8*^*wdl*^ (20 Hz)	3 C57BLKS/J (20 Hz) vs. 3 *Car8*^*wdl*^ (20 Hz)	127.6 ± 4.8% (C57BLKS/J, 20 Hz) vs. 125.3 ± 4.6% (*Car8*^*wdl*^, 20 Hz)	98,657 ± 3,682 μm^2^ (C57BLKS/J, 20 Hz) vs. 96,886 ± 3,590 μm^2^ (*Car8*^*wdl*^, 20 Hz)	p=0.9896	Yes	3E
		Tukey’s multiple comparisons test	31 C57BLKS/J (0 Hz), 31 *Car8*^*wdl*^ (0 Hz); 30 C57BLKS/J (20 Hz), 30 *Car8*^*wdl*^ (20 Hz)	3 *Car8*^*wdl*^ (0 Hz) vs. 3 *Car8*^*wdl*^ (20 Hz)	138.3 ± 5.1% (*Car8*^*wdl*^, 0 Hz) vs. 125.3 ± 4.6% (*Car8*^*wdl*^, 20 Hz)	106,942 ± 3,969 μm^2^ (*Car8*^*wdl*^, 0 Hz) vs. 96,886 ± 3,590 μm^2^ (*Car8*^*wdl*^, 20 Hz)	p=0.2900	No	3E
Mitochondrial aspect ratio	Yes; % Untreated C57BLKS/J Controls	Two-way ANOVA	31 C57BLKS/J (0 Hz), 31 *Car8*^*wdl*^ (0 Hz); 30 C57BLKS/J (20 Hz), 30 *Car8*^*wdl*^ (20 Hz)	3 C57BLKS/J (0 Hz), 3 *Car8*^*wdl*^ (0 Hz); 3 C57BLKS/J (20 Hz), 3 *Car8*^*wdl*^ (20 Hz)	97.44 ± 1.58% (C57BLKS/J, 0 Hz) vs. 98.24 ± 1.36% (C57BLKS/J, 20 Hz) vs. 96.16 ± 1.21% (*Car8*^*wdl*^, 0 Hz) vs. 96.87 ± 1.24% (*Car8*^*wdl*^, 20 Hz)	1.830 ± 0.030 (C57BLKS/J, 0 Hz) vs. 1.845 ± 0.026 (C57BLKS/J, 20 Hz) vs. 1.806 ± 0.022 (*Car8*^*wdl*^, 0 Hz) vs. 1.820 ± 0.023 (*Car8*^*wdl*^, 20 Hz)	Genotype, p=0.3299; Treatment, p=0.5784; Genotype x Treatment, p=0.9753	No	3F
Total MERC number	Yes; % Untreated C57BLKS/J Controls	Two-way ANOVA	31 C57BLKS/J (0 Hz), 31 *Car8*^*wdl*^ (0 Hz); 30 C57BLKS/J (20 Hz), 30 *Car8*^*wdl*^ (20 Hz)	3 C57BLKS/J (0 Hz), 3 *Car8*^*wdl*^ (0 Hz); 3 C57BLKS/J (20 Hz), 3 *Car8*^*wdl*^ (20 Hz)	100.50 ± 8.12% (C57BLKS/J, 0 Hz) vs. 76.72 ± 5.43% (C57BLKS/J, 20 Hz) vs. 95.67 ± 5.05% (*Car8*^*wdl*^, 0 Hz) vs. 138.20 ± 8.63% (*Car8*^*wdl*^, 20 Hz)	153.80 ± 12.43 MERCs (C57BLKS/J, 0 Hz) vs. 117.50 ± 8.31 MERCs (C57BLKS/J, 20 Hz) vs. 146.50 ± 7.73 MERCs (*Car8*^*wdl*^, 0 Hz) vs. 211.60 ± 13.21 MERCs (*Car8*^*wdl*^, 20 Hz)	Genotype, p<0.0001; Treatment, p=0.1814; Genotype x Treatment, p<0.0001	Yes/No	4A
		Tukey’s multiple comparisons test	31 C57BLKS/J (0 Hz) vs. 30 C57BLKS/J (20 Hz)	3 C57BLKS/J (0 Hz) vs. 3 C57BLKS/J (20 Hz)	100.50 ± 8.12% (C57BLKS/J, 0 Hz) vs. 76.72 ± 5.43% (C57BLKS/J, 20 Hz)	153.80 ± 12.43 MERCs (C57BLKS/J, 0 Hz) vs. 117.50 ± 8.31 MERCs (C57BLKS/J, 20 Hz)	p=0.0815	No	4A
		Tukey’s multiple comparisons test	31 C57BLKS/J (0 Hz) vs. 31 *Car8*^*wdl*^ (0 Hz)	3 C57BLKS/J (0 Hz) vs. 3 *Car8*^*wdl*^ (0 Hz)	100.50 ± 8.12% (C57BLKS/J, 0 Hz) vs. 95.67 ± 5.05% (*Car8*^*wdl*^, 0 Hz)	153.80 ± 12.43 MERCs (C57BLKS/J, 0 Hz) vs. 146.50 ± 7.73 MERCs (*Car8*^*wdl*^, 0 Hz)	p=0.9611	No	4A
		Tukey’s multiple comparisons test	31 C57BLKS/J (0 Hz) vs. 30 *Car8*^*wdl*^ (20 Hz)	3 C57BLKS/J (0 Hz) vs. 3 *Car8*^*wdl*^ (20 Hz)	100.50 ± 8.12% (C57BLKS/J, 0 Hz) vs. 138.20 ± 8.63% (*Car8*^*wdl*^, 20 Hz)	153.80 ± 12.43 MERCs (C57BLKS/J, 0 Hz) vs. 211.60 ± 13.21 MERCs (*Car8*^*wdl*^, 20 Hz)	p=0.0012	Yes	4A
		Tukey’s multiple comparisons test	31 *Car8*^*wdl*^ (0 Hz) vs. 30 C57BLKS/J (20 Hz)	3 *Car8*^*wdl*^ (0 Hz) vs. 3 C57BLKS/J (20 Hz)	76.72 ± 5.43% (C57BLKS/J, 20 Hz) vs. 95.67 ± 5.05% (*Car8*^*wdl*^, 0 Hz)	117.50 ± 8.31 MERCs (C57BLKS/J, 20 Hz) vs. 146.50 ± 7.73 MERCs (*Car8*^*wdl*^, 0 Hz)	p=0.2256	No	4A
		Tukey’s multiple comparisons test	30 C57BLKS/J (20 Hz) vs. 30 *Car8*^*wdl*^ (20 Hz)	3 C57BLKS/J (20 Hz) vs. 3 *Car8*^*wdl*^ (20 Hz)	76.72 ± 5.43% (C57BLKS/J, 20 Hz) vs. 138.20 ± 8.63% (*Car8*^*wdl*^, 20 Hz)	117.50 ± 8.31 MERCs (C57BLKS/J, 20 Hz) vs. 211.60 ± 13.21 MERCs (*Car8*^*wdl*^, 20 Hz)	p<0.0001	Yes	4A
		Tukey’s multiple comparisons test	31 *Car8*^*wdl*^ (0 Hz) vs. 30 *Car8*^*wdl*^ (20 Hz)	3 *Car8*^*wdl*^ (0 Hz) vs. 3 *Car8*^*wdl*^ (20 Hz)	95.67 ± 5.05% (*Car8*^*wdl*^, 0 Hz) vs. 138.20 ± 8.63% (*Car8*^*wdl*^, 20 Hz)	146.50 ± 7.73 MERCs (*Car8*^*wdl*^, 0 Hz) vs. 211.60 ± 13.21 MERCs (*Car8*^*wdl*^, 20 Hz)	p=0.0002	Yes	4A
% Mitochondria with MERCs	None	Chi-Squared Test	31 C57BLKS/J (0 Hz), 31 *Car8*^*wdl*^ (0 Hz); 30 C57BLKS/J (20 Hz), 30 *Car8*^*wdl*^ (20 Hz)	3 C57BLKS/J (0 Hz), 3 *Car8*^*wdl*^ (0 Hz); 3 C57BLKS/J (20 Hz), 3 *Car8*^*wdl*^ (20 Hz)	88.11 ± 5.23% (C57BLKS/J, 0 Hz) vs. 86.70 ± 2.29% (C57BLKS/J, 20 Hz) vs. 93.59 ± 0.94% (*Car8*^*wdl*^, 0 Hz) vs. 95.43 ± 1.37% (*Car8*^*wdl*^, 20 Hz)	No normalization performed	p=0.1066	No	4B
MERC/mitochondria	Yes; % Untreated C57BLKS/J Controls	Two-way ANOVA	31 C57BLKS/J (0 Hz), 31 *Car8*^*wdl*^ (0 Hz); 30 C57BLKS/J (20 Hz), 30 *Car8*^*wdl*^ (20 Hz)	3 C57BLKS/J (0 Hz), 3 *Car8*^*wdl*^ (0 Hz); 3 C57BLKS/J (20 Hz), 3 *Car8*^*wdl*^ (20 Hz)	123.40 ± 7.10% (C57BLKS/J, 0 Hz) vs. 109.90 ± 5.50% (C57BLKS/J, 20 Hz) vs. 143.00 ± 4.53% (*Car8*^*wdl*^, 0 Hz) vs. 154.90 ± 5.01% (*Car8*^*wdl*^, 20 Hz)	2.148 ± 0.124 MERC/mito (C57BLKS/J, 0 Hz) vs. 1.912 ± 0.096 MERC/mito (C57BLKS/J, 20 Hz) vs. 2.488 ± 0.079 MERC/mito (*Car8*^*wdl*^, 0 Hz) vs. 2.696 ± 0.087 MERC/mito (*Car8*^*wdl*^, 20 Hz)	Genotype, p<0.0001; Treatment, p=0.8843; Genotype x Treatment, p=0.0256	Yes/No	4C
		Tukey’s multiple comparisons test	31 C57BLKS/J (0 Hz) vs. 30 C57BLKS/J (20 Hz)	3 C57BLKS/J (0 Hz) vs. 3 C57BLKS/J (20 Hz)	123.40 ± 7.10% (C57BLKS/J, 0 Hz) vs. 109.90 ± 5.50% (C57BLKS/J, 20 Hz)	2.148 ± 0.124 MERC/mito (C57BLKS/J, 0 Hz) vs. 1.912 ± 0.096 MERC/mito (C57BLKS/J, 20 Hz)	p=0.3271	No	4C
		Tukey’s multiple comparisons test	31 C57BLKS/J (0 Hz) vs. 31 *Car8*^*wdl*^ (0 Hz)	3 C57BLKS/J (0 Hz) vs. 3 *Car8*^*wdl*^ (0 Hz)	123.40 ± 7.10% (C57BLKS/J, 0 Hz) vs. 143.00 ± 4.53% (*Car8*^*wdl*^, 0 Hz)	2.148 ± 0.124 MERC/mito (C57BLKS/J, 0 Hz) vs. 2.488 ± 0.079 MERC/mito (*Car8*^*wdl*^, 0 Hz)	p=0.0690	No	4C
		Tukey’s multiple comparisons test	31 C57BLKS/J (0 Hz) vs. 30 *Car8*^*wdl*^ (20 Hz)	3 C57BLKS/J (0 Hz) vs. 3 *Car8*^*wdl*^ (20 Hz)	123.40 ± 7.10% (C57BLKS/J, 0 Hz) vs. 154.90 ± 5.01% (*Car8*^*wdl*^, 20 Hz)	2.148 ± 0.124 MERC/mito (C57BLKS/J, 0 Hz) vs. 2.696 ± 0.087 MERC/mito (*Car8*^*wdl*^, 20 Hz)	p=0.0008	Yes	4C
		Tukey’s multiple comparisons test	31 *Car8*^*wdl*^ (0 Hz) vs. 30 C57BLKS/J (20 Hz)	3 *Car8*^*wdl*^ (0 Hz) vs. 3 C57BLKS/J (20 Hz)	109.90 ± 5.50% (C57BLKS/J, 20 Hz) vs. 143.00 ± 4.53% (*Car8*^*wdl*^, 0 Hz)	1.912 ± 0.096 MERC/mito (C57BLKS/J, 20 Hz) vs. 2.488 ± 0.079 MERC/mito (*Car8*^*wdl*^, 0 Hz)	p=0.0004	Yes	4C
		Tukey’s multiple comparisons test	30 C57BLKS/J (20 Hz) vs. 30 *Car8*^*wdl*^ (20 Hz)	3 C57BLKS/J (20 Hz) vs. 3 *Car8*^*wdl*^ (20 Hz)	109.90 ± 5.50% (C57BLKS/J, 20 Hz) vs. 154.90 ± 5.01% (*Car8*^*wdl*^, 20 Hz)	1.912 ± 0.096 MERC/mito (C57BLKS/J, 20 Hz) vs. 2.696 ± 0.087 MERC/mito (*Car8*^*wdl*^, 20 Hz)	p<0.0001	Yes	4C
		Tukey’s multiple comparisons test	31 *Car8*^*wdl*^ (0 Hz) vs. 30 *Car8*^*wdl*^ (20 Hz)	3 *Car8*^*wdl*^ (0 Hz) vs. 3 *Car8*^*wdl*^ (20 Hz)	143.00 ± 4.53% (*Car8*^*wdl*^, 0 Hz) vs. 154.90 ± 5.01% (*Car8*^*wdl*^, 20 Hz)	2.488 ± 0.079 MERC/mito (*Car8*^*wdl*^, 0 Hz) vs. 2.696 ± 0.087 MERC/mito (*Car8*^*wdl*^, 20 Hz)	p=0.4432	No	4C
Untreated *Car8*^*wdl*^ correlation matrices	Yes; % Untreated C57BLKS/J Controls	Pearson correlation coefficient tests	15 *Car8*^*wdl*^	3 *Car8*^*wdl*^	N/A	N/A	Density vs. Area / Area vs. Density: p=0.637	No	5A
		Pearson correlation coefficient tests	15 *Car8*^*wdl*^	3 *Car8*^*wdl*^	N/A	N/A	Density vs. Total / Total vs. Density: p=0.001	Yes	5A
		Pearson correlation coefficient tests	15 *Car8*^*wdl*^	3 *Car8*^*wdl*^	N/A	N/A	Density vs. Distribution / Distribution vs. Density: p=0.480	No	5A
		Pearson correlation coefficient tests	15 *Car8*^*wdl*^	3 *Car8*^*wdl*^	N/A	N/A	Area vs. Total / Total vs. Area: p=0.350	No	5A
		Pearson correlation coefficient tests	15 *Car8*^*wdl*^	3 *Car8*^*wdl*^	N/A	N/A	Area vs. Distribution / Distribution vs. Area: p=0.032	Yes	5A
		Pearson correlation coefficient tests	15 *Car8*^*wdl*^	3 *Car8*^*wdl*^	N/A	N/A	Total vs. Distribution / Distribution vs. Total: p=0.084	Yes	5A
Sham (0 Hz) *Car8*^*wdl*^ correlation matrices	Yes; % Untreated C57BLKS/J Controls	Pearson correlation coefficient tests	31 *Car8*^*wdl*^	3 *Car8*^*wdl*^	N/A	N/A	Density vs. Area / Area vs. Density: p=0.484	No	5A
		Pearson correlation coefficient tests	31 *Car8*^*wdl*^	3 *Car8*^*wdl*^	N/A	N/A	Density vs. Total / Total vs. Density: p=0.800	No	5A
		Pearson correlation coefficient tests	31 *Car8*^*wdl*^	3 *Car8*^*wdl*^	N/A	N/A	Density vs. Distribution / Distribution vs. Density: p=0.249	No	5A
		Pearson correlation coefficient tests	31 *Car8*^*wdl*^	3 *Car8*^*wdl*^	N/A	N/A	Area vs. Total / Total vs. Area: p=0.816	No	5A
		Pearson correlation coefficient tests	31 *Car8*^*wdl*^	3 *Car8*^*wdl*^	N/A	N/A	Area vs. Distribution / Distribution vs. Area: p=0.076	No	5A
		Pearson correlation coefficient tests	31 *Car8*^*wdl*^	3 *Car8*^*wdl*^	N/A	N/A	Total vs. Distribution / Distribution vs. Total: p<0.0001	Yes	5A
Stimulated (20 Hz) *Car8*^*wdl*^ correlation matrices	Yes; % Untreated C57BLKS/J Controls	Pearson correlation coefficient tests	30 *Car8*^*wdl*^	3 *Car8*^*wdl*^	N/A	N/A	Density vs. Area / Area vs. Density: p=0.658	No	5A
		Pearson correlation coefficient tests	30 *Car8*^*wdl*^	3 *Car8*^*wdl*^	N/A	N/A	Density vs. Total / Total vs. Density: p=0.745	No	5A
		Pearson correlation coefficient tests	30 *Car8*^*wdl*^	3 *Car8*^*wdl*^	N/A	N/A	Density vs. Distribution / Distribution vs. Density: p=0.828	No	5A
		Pearson correlation coefficient tests	30 *Car8*^*wdl*^	3 *Car8*^*wdl*^	N/A	N/A	Area vs. Total / Total vs. Area: p=0.079	No	5A
		Pearson correlation coefficient tests	30 *Car8*^*wdl*^	3 *Car8*^*wdl*^	N/A	N/A	Area vs. Distribution / Distribution vs. Area: p=0.715	No	5A
		Pearson correlation coefficient tests	30 *Car8*^*wdl*^	3 *Car8*^*wdl*^	N/A	N/A	Total vs. Distribution / Distribution vs. Total: p<0.0001	Yes	5A
K-means clustering	Yes; % Untreated C57BLKS/J Controls	Two-way ANOVA	20 Cluster 1 vs. 10 Cluster 2 vs. 31 Cluster 3	2 Cluster 1 vs. 1 Cluster 2 vs. 3 Cluster 3	86.914 ± 5.301% (Density, Cluster 1 vs. 106.921 ± 16.206% (Density, Cluster 2 vs. 69.238 ± 2.668% (Density, Cluster 3) vs. 114.626 ± 4.899% (Area, Cluster 1) vs. 146.674 ± 5.568% (Area, Cluster 2) vs. 138.314 ± 5.133% (Area, Cluster 3) vs. 136.731 ± 11.554% (Total, Cluster 1) vs. 141.139 ± 12.440% (Total, Cluster 2) vs. 95.672 ± 5.046% (Total, Cluster 3); 149.715 ± 6.384% (Distribution, Cluster 1) vs. 165.274 ± 7.246% (Distribution, Cluster 2) vs. 142.990 ± 4.527% (Distribution, Cluster 3)		Cluster, p<0.0001; Characteristic, p<0.0001; Cluster x Characteristic, p<0.0001	Yes	5C
		Tukey’s multiple comparisons test	20 Cluster 1 vs. 10 Cluster 2	2 Cluster 1 vs. 1 Cluster 2	86.914 ± 5.301% (Density, Cluster 1) vs. 106.921 ± 16.206% (Density, Cluster 2)	0.9920 ± 0.0605 mito/PC (Density, Cluster 1) vs. 1.220 ± 0.1850 (Density, Cluster 2)	p=0.8434	No	5C
		Tukey’s multiple comparisons test	20 Cluster 1 vs. 31 Cluster 3	2 Cluster 1 vs. 3 Cluster 3	86.914 ± 5.301% (Density, Cluster 1) vs. 69.238 ± 2.668% (Density, Cluster 3)	0.9920 ± 0.0605 mito/PC (Density, Cluster 1) vs. 0.7902 ± 0.0305 mito/PC (Density, Cluster 3)	p=0.6421	No	5C
		Tukey’s multiple comparisons test	10 Cluster 2 vs. 31 Cluster 3	1 Cluster 2 vs. 3 Cluster 3	106.921 ± 16.206% (Density, Cluster 2) vs. 69.238 ± 2.668% (Density, Cluster 3)	1.220 ± 0.1850 (Density, Cluster 2) vs. 0.7902 ± 0.0305 mito/PC (Density, Cluster 3)	p=0.0284	Yes	5C
		Tukey’s multiple comparisons test	20 Cluster 1 vs. 10 Cluster 2	2 Cluster 1 vs. 1 Cluster 2	114.626 ± 4.899% (Area, Cluster 1) vs. 146.674 ± 5.568% (Area, Cluster 2)	88,627 ± 3,788 μm^2^ (Area, Cluster 1) vs. 113,406 ± 4,305 μm^2^ (Area, Cluster 2)	p=0.1939	No	5C
		Tukey’s multiple comparisons test	20 Cluster 1 vs. 31 Cluster 3	2 Cluster 1 vs. 3 Cluster 3	114.626 ± 4.899% (Area, Cluster 1) vs. 138.314 ± 5.133% (Area, Cluster 3)	88,627 ± 3,788 μm^2^ (Area, Cluster 1) vs. 106,942 ± 3,969 μm^2^ (Area, Cluster 3)	p=0.1961	No	5C
		Tukey’s multiple comparisons test	10 Cluster 2 vs. 31 Cluster 3	1 Cluster 2 vs. 3 Cluster 3	146.674 ± 5.568% (Area, Cluster 2) vs. 138.314 ± 5.133% (Area, Cluster 3)	113,406 ± 4,305 μm^2^ (Area, Cluster 2) vs. 106,942 ± 3,969 μm^2^ (Area, Cluster 3)	p=0.9998	No	5C
		Tukey’s multiple comparisons test	20 Cluster 1 vs. 10 Cluster 2	2 Cluster 1 vs. 1 Cluster 2	136.731 ± 11.554% (Total, Cluster 1) vs. 141.139 ± 12.440% (Total, Cluster 2)	209.40 ± 17.69 MERCs (Total, Cluster 1) vs. 216.10 ± 19.05 MERCs (Total, Cluster 2)	p>0.9999	No	5C
		Tukey’s multiple comparisons test	20 Cluster 1 vs. 31 Cluster 3	2 Cluster 1 vs. 3 Cluster 3	136.731 ± 11.554% (Total, Cluster 1) vs. 95.672 ± 5.046% (Total, Cluster 3)	209.40 ± 17.69 MERCs (Total, Cluster 1) vs. 146.50 ± 7.73 MERCs (Total, Cluster 3)	p=0.0002	Yes	5C
		Tukey’s multiple comparisons test	10 Cluster 2 vs. 31 Cluster 3	1 Cluster 2 vs. 3 Cluster 3	141.139 ± 12.440% (Total, Cluster 2) vs. 95.672 ± 5.046% (Total, Cluster 3)	216.10 ± 19.05 MERCs (Total, Cluster 2) vs. 146.50 ± 7.73 MERCs (Total, Cluster 3)	p=0.0022	Yes	5C
		Tukey’s multiple comparisons test	20 Cluster 1 vs. 10 Cluster 2	2 Cluster 1 vs. 1 Cluster 2	149.715 ± 6.384% (Distribution, Cluster 1) vs. 165.274 ± 7.246% (Distribution, Cluster 2)	2.605 ± 0.111 MERC/mito (Distribution, Cluster 1) vs. 2.876 ± 0.126 MERC/mito (Distribution, Cluster 2)	p=0.9711	No	5C
		Tukey’s multiple comparisons test	20 Cluster 1 vs. 31 Cluster 3	2 Cluster 1 vs. 3 Cluster 3	149.715 ± 6.384% (Distribution, Cluster 1) vs. 142.990 ± 4.527% (Distribution, Cluster 3)	2.605 ± 0.111 MERC/mito (Distribution, Cluster 1) vs. 2.488 ± 0.079 MERC/mito (Distribution, Cluster 3)	p=0.9997	No	5C
		Tukey’s multiple comparisons test	10 Cluster 2 vs. 31 Cluster 3	1 Cluster 2 vs. 3 Cluster 3	165.274 ± 7.246% (Distribution, Cluster 2) vs. 142.990 ± 4.527% (Distribution, Cluster 3)	2.876 ± 0.126 MERC/mito (Distribution, Cluster 2) vs. 2.488 ± 0.079 MERC/mito (Distribution, Cluster 3)	p=0.6505	No	5C
